# Analysis of Upper Gastrointestinal Adverse Events Associated with Oral Anticoagulants and Potential Drug Interactions with Cardiovascular Drugs: Exploratory Study Using FDA Adverse Event Reporting System

**DOI:** 10.3390/ph18091311

**Published:** 2025-09-01

**Authors:** Seunghyun Cheon, Jiyeon Park, Dosol Oh, Young Seo Kim, Jee-Eun Chung

**Affiliations:** 1 College of Pharmacy, Institute of Pharmaceutical Science and Technology, Hanyang University, Ansan 15588, Republic of Korea; cshh1005@hanyang.ac.kr (S.C.);; 2 Department of Neurology, Hanyang University Hospital, College of Medicine, Hanyang University, Seoul 04763, Republic of Korea; kimys1@hanyang.ac.kr

**Keywords:** oral anticoagulant, upper gastrointestinal adverse events, disproportionality analysis, drug interaction, FDA Adverse Event Reporting System

## Abstract

**Background:** This study aimed to evaluate the risk of upper gastrointestinal (UGI) adverse events (AEs) associated with oral anticoagulants (OACs) and identify potential interactions with cardiovascular (CV) drugs. **Methods:** Individual case safety reports (ICSRs) from the FDA Adverse Event Reporting System from July 2014 to December 2023 were analyzed. Dataset I was constructed to assess the associations between OACs and UGI AEs using disproportionality analysis. Dataset Ⅱ included OAC-related ICSRs to explore potential interactions with CV drugs through logistic regression. Positive signals were defined as potential associations identified by disproportionality analysis metrics, such as reporting odds ratios (RORs) or adjusted RORs (aRORs) accounting for confounders. **Results:** Dataset I included 12,905,290 ICSRs, and a positive signal for dabigatran was detected with an ROR of 1.19 (95% CI, 1.13–1.25). A total of 364,044 OAC-related ICSRs were included in dataset II. At the pharmacologic drug class level, several positive signals were identified, represented as aRORs with 95% CIs: for warfarin, amiodarone analogs (1.22; 1.04–1.43); for apixaban, angiotensin-converting enzyme inhibitors (1.34; 1.24–1.45), angiotensin receptor blockers (1.23; 1.14–1.33), dihydropyridine calcium channel blockers (1.30; 1.21–1.41), and digitalis glycosides (1.72; 1.49–2.00); and for edoxaban, angiotensin receptor blockers (1.88; 1.48–2.37), amiodarone analogs (1.73; 1.06–2.85), and anti-platelets (1.56; 1.20–2.03). No signals were observed for rivaroxaban or dabigatran. At the individual drug level, 62 OAC-CV pairs were identified as having potential interactions. **Conclusions:** Drug-specific interaction profiles should be considered to ensure safe and personalized use of OACs in clinical practice.

## 1. Introduction

Upper gastrointestinal (UGI) adverse events (AEs), such as abdominal discomfort, indigestion, ulcer-like symptoms, and gastritis, are frequently reported with a wide range of medications. While UGI AEs are generally not associated with life-threatening or clinically serious outcomes, they have the potential to reduce medication adherence and lead to discontinuation of treatment [1]. This, in turn, would negatively impact the overall treatment effectiveness. The risk of drug-induced UGI AEs may be increased in patients undergoing polypharmacy [2], and it is particularly pronounced in elderly patients due to physiological changes in the gastrointestinal (GI) tract associated with aging [3].

Among medications associated with UGI AEs, direct oral anticoagulants (DOACs) have recently garnered attention for their potential to cause these AEs. DOACs—including dabigatran, rivaroxaban, apixaban, and edoxaban—were introduced as alternatives to warfarin, aiming to overcome several of its key limitations, such as a narrow therapeutic range, extensive food and drug interactions, and the need for regular monitoring [4]. In current clinical practice, DOACs are the preferred options for several conditions requiring anticoagulation therapy, including atrial fibrillation and venous thromboembolism, due to their predictable pharmacokinetics and simplified management compared to warfarin [5,6]. Despite their benefits over warfarin, UGI safety has emerged as a growing concern based on findings from both clinical trials and post-marketing surveillance. This has prompted the US FDA to include the UGI risks in the official labeling of each DOAC. Among the DOACs, dabigatran has shown a relatively high frequency of UGI AEs. In a clinical trial (RE-LY), approximately 35% of patients who received 150 mg of dabigatran reported UGI symptoms such as abdominal pain, gastroesophageal reflux disease (GERD), esophagitis, and ulcers [7]. Post-marketing surveillance has suggested a potential association between dabigatran and the development of esophageal ulcers [8]. Rivaroxaban has similarly demonstrated GI events in clinical trials. In the UNIVERSE study, approximately 12.5% of patients experienced gastroenteritis [9], and in the EINSTEIN-DVT study, 2.7% of patients complained of abdominal pain [10]. For edoxaban, abdominal pain has been identified as an additional AE in post-marketing surveillance [8]. Although apixaban is not labeled for GI AEs other than nausea, approximately 10% of patients initiating apixaban were prescribed proton pump inhibitors (PPIs) within 6 months, possibly indicating underlying UGI tolerance [11]. Given these safety concerns regarding UGI AEs associated with DOACs, clinicians often prescribe acid-suppressive agents or switch to alternative OACs [11,12]. In a previously registered study, dyspepsia accounted for 6.6% of all causes of dabigatran discontinuation, including socioeconomic factors, and 18.9% when considering only adverse events. Additionally, PPI use was identified as a strong risk factor for discontinuation. These findings support the notion that UGI tolerability influences patient adherence and impacts clinicians’ prescribing behavior and monitoring strategies [13].

In real-world settings, patients prescribed DOACs are often concurrently treated with a variety of cardiovascular (CV) drugs, such as beta-adrenergic receptor blockers (BBs), angiotensin-converting enzyme (ACE) inhibitors, diuretics, and hydroxymethylglutaryl-CoA reductase inhibitors (statins) [14]. In a study conducted by Honda et al., more than 80% of patients with atrial fibrillation on DOAC therapy had comorbid CV conditions, and over 75% were concomitantly taking five or more medications [15]. Although drug–drug interactions involving DOACs have been extensively studied, they have primarily focused on bleeding risk and interactions with cytochrome P450 enzyme (CYP) or P-glycoprotein (P-gp) inhibitors [16,17,18]. In contrast, the potential impact of such interactions on UGI AEs remains largely unexplored. Therefore, this study aims to investigate the risk of UGI AEs associated with DOAC use and to assess the potential for drug–drug interactions that contribute to these events, using data from the FDA Adverse Event Reporting System (FAERS).

## 2. Results

### 2.1. Dataset Construction

**Figure 1:**
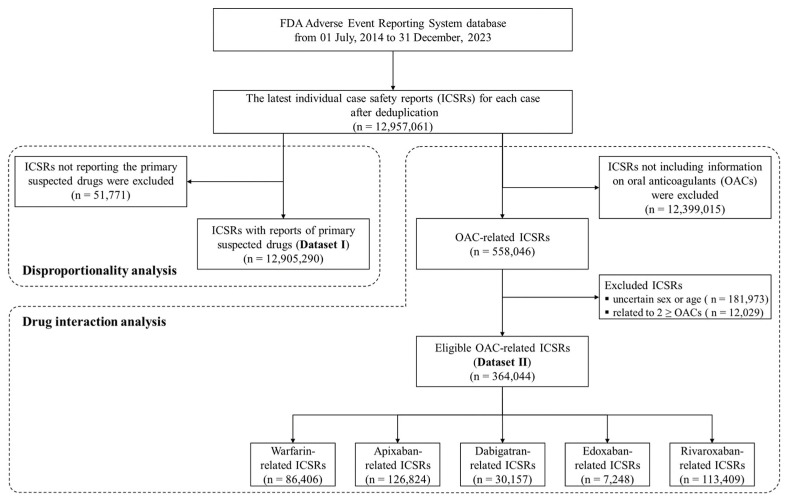
Flowchart for constructing datasets in the study.

From the FAERS database, covering 1 July 2014 to 31 December 2023, a total of 12,957,061 individual case safety reports (ICSRs) were identified after removing duplicates and retaining only the latest report for each case. Among these ICSRs, 12,905,290 ICSRs reported primary suspected drugs (dataset I). Subsequently, the other dataset encompassing 558,046 ICSRs was identified as involving oral anticoagulants (OACs). After excluding 12,029 ICSRs related to two or more OACs and 181,973 ICSRs with missing information on sex or age, 364,044 ICSRs were included as eligible for analysis (dataset II). Following the categorization of dataset II according to the OAC, it was determined that a warfarin sub-dataset was present in a total of 86,406 ICSRs, an apixaban sub-dataset in 126,824 ICSRs, a dabigatran sub-dataset in 30,157 ICSRs, an edoxaban sub-dataset in 7248 ICSRs, and a rivaroxaban sub-dataset in 113,409 ICSRs. The process of constructing these datasets is illustrated in Figure 1.

### 2.2. Disproportionality Analysis for OAC-Related UGI AEs

In dataset Ⅰ, there were 24,201, 122,471, 34,854, 6121, and 95,734 ICSRs reporting warfarin, apixaban, dabigatran, edoxaban, and rivaroxaban as primary suspected drugs, respectively. Only 3.2% of OAC-related ICSRs reported UGI AEs, with dabigatran showing the highest frequency at 4.7%. The results of the four metrics—the reporting odds ratio (ROR), proportional reporting ratio (PRR), information component (IC), and empirical Bayesian geometric mean (EBGM)—obtained from the disproportionality analysis are shown in Table 1. Only dabigatran showed statistically significant results for the ROR, PRR, and IC_025_.

### 2.3. Descriptive Analysis for OAC-Related ICSRs

The demographic characteristics and co-medication information of the ICSRs in the five sub-datasets derived from dataset II are summarized in Table 2. The utilization of OACs was prevalent among individuals aged 40 years and older; however, there were notable variations in the age distribution of each OAC. Whereas warfarin and rivaroxaban demonstrated extensive utilization across all age groups, with a relatively high proportion of usage observed in the 40–64 age group, apixaban, dabigatran, and edoxaban accounted for more than half of those aged 75 years or older.

Co-medication was identified at a high rate among OAC users, with more than 70% of all users taking additional medications. Polypharmacy was particularly prevalent among warfarin and edoxaban users, at approximately 92% and 90%, respectively. The most frequently reported co-administered CV drug class was BB, used by between 24.2% and 41.4% of each OAC group. This was followed by diuretics (18.7–37.4%), statins (19.1–27.3%), anti-platelets (12.7–27.9%), angiotensin receptor blockers (ARBs; 13.2–22.6%), and ACE inhibitors (10.7–16.6%). Additionally, PPIs were commonly used for acid suppression and prevention of UGI AEs, with a prevalence ranging from 17.0% to 34.5%. The proportion of ICSRs receiving acid-suppressive therapy, including PPI use, was nearly twice as high with edoxaban at 39.7% compared to with other OACs.

Approximately 5% of OAC-related ICSRs involved UGI AEs: 5569 ICSRs (6.4%) in the warfarin group, 5735 (4.5%) in the apixaban group, 1698 (5.6%) in the dabigatran group, 456 (6.3%) in the edoxaban group, and 6231 (5.5%) in the rivaroxaban group.

### 2.4. Drug Interaction Analysis

For each OAC sub-dataset, exact matching was independently performed 14 times based on exposure to either a positive control or drugs of interest, resulting in 70 separate matched datasets. The matched datasets comprised approximately 79,000 ICSRs for warfarin (min, 77,636; max, 79,898), 84,000 for apixaban (min, 82,522; max, 84,170), 20,000 for dabigatran (min, 19,706; max, 20,446), 6000 for edoxaban (min, 5799; max, 6377), and 81,000 for rivaroxaban (min, 79,988; max, 81,634).

#### 2.4.1. Drug Interaction of OACs and Positive Control

Within the matched OAC-related ICSRs, the concomitant use of non-steroidal anti-inflammatory drugs (NSAIDs) was associated with a significantly increased risk of UGI AEs across all OAC types in the univariate analysis (Table 3). The crude reporting odds ratios (cRORs) indicated a 26% to 148% elevation in UGI AE risk. However, after adjusting for age, sex, and co-medication, dabigatran showed no statistically significant increase in risk (adjusted reporting odds ratio [aROR], 1.21; 95% CI, 0.97–1.49), suggesting a negligible drug interaction effect. In contrast, the increased risk remained significant for the other OACs: the aROR was 1.39 (95% CI, 1.25–1.54) for warfarin, 1.95 (95% CI, 1.78–2.14) for apixaban, 1.77 (95% CI, 1.29–2.40) for edoxaban, and 1.86 (95% CI, 1.71–2.03) for rivaroxaban.

#### 2.4.2. Drug Interaction of OACs and Pharmacokinetic Modulators

In the matched dataset comparing CYP inhibitor users and non-users, concomitant use of CYP inhibitors ranged from 24.3% to 31.5% depending on the type of OAC. A total of 57 CYP inhibitors were evaluated, with amiodarone (24.7%), clopidogrel (23.7%), and diltiazem (18.5%) being the most frequently reported. In a separately matched dataset for P-gp inhibitors, approximately 16.8% of ICSRs involved P-gp use, with the highest proportion observed in the dabigatran group (25.6%) and the lowest in the rivaroxaban group (14.0%). The most commonly reported P-gp inhibitors were amiodarone (39.7%), digoxin (34.0%), and diltiazem (29.7%).

**Figure 2:**
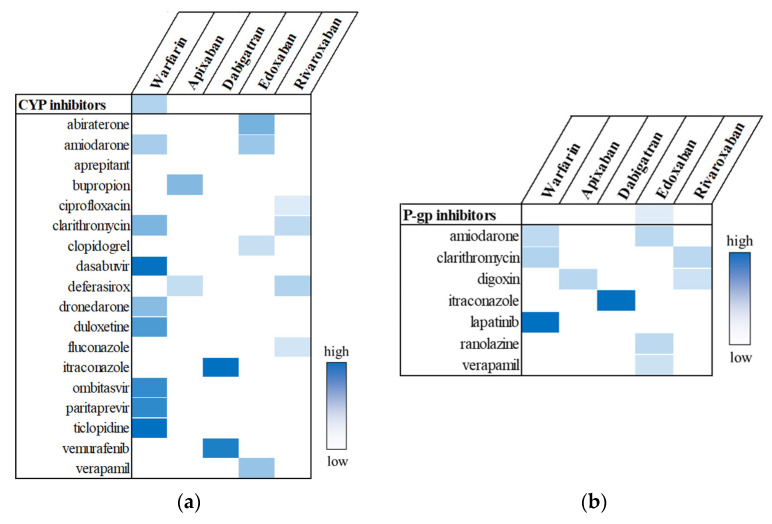
Potential drug interactions between OACs and CYP inhibitors (**a**) or P-gp inhibitors (**b**). Statistically significant signals are highlighted in color, with greater intensity indicating higher aROR values.

As shown in Figure 2a, the pooled analysis, which evaluated the use of the CYP inhibitor class in relation to UGI AE risk across each OAC, identified a significant signal only for warfarin (aROR, 1.19; 95% CI, 1.11–1.27). In individual drug analyses, six CYP inhibitors—clarithromycin, dasabuvir, duloxetine, ombitasvir, paritaprevir, and ticlopidine—were positively associated with warfarin. Additionally, one CYP inhibitor was associated with apixaban, two with dabigatran, and four each with edoxaban and rivaroxaban. On the other hand, with regard to P-gp inhibitors, the use of the P-gp inhibitor group was not significantly associated with UGI AEs in any OAC group (Figure 2b). Nine OAC–P-gp inhibitor pairs showed positive signals. The strongest association was observed between dabigatran and itraconazole (aROR, 9.07; 95% CI, 3.09–25.60), although this finding was based on only 16 cases. The results for individual drugs are listed in Supplementary Tables S1–S5.

#### 2.4.3. Drug Interaction of OACs and Cardiovascular Drugs

To evaluate potential UGI AE risks associated with concomitant use of OACs and CV drugs, 11 separate matched datasets were constructed for each CV drug class. These datasets were developed independently, ensuring that each analysis was conducted with a mutually exclusive and statistically independent dataset.

A total of 139 CV drugs were included, encompassing BBs, ACE inhibitors, ARBs, dihydropyridine calcium channel blockers (DHP-CCBs), non-dihydropyridine calcium channel blockers (NDHP-CCBs), diuretics, statins, other lipid-lowering agents, amiodarone analogs, digitalis glycosides, and anti-platelets. The most frequently reported medications in each drug class were as follows: metoprolol (41.2%) for BBs, lisinopril (43.3%) for ACE inhibitors, losartan (36.0%) for ARBs, amlodipine (80.2%) for DHP-CCBs, diltiazem (79.9%) for NDHP-CCBs, furosemide (63.0%) for diuretics, atorvastatin (50.0%) for statins, ezetimibe (46.3%) for other lipid-lowering agents, amiodarone (91.8%) for amiodarone analogs, digoxin (93.4%) for digitalis glycosides, and aspirin (85.5%) for anti-platelets.

**Figure 3:**
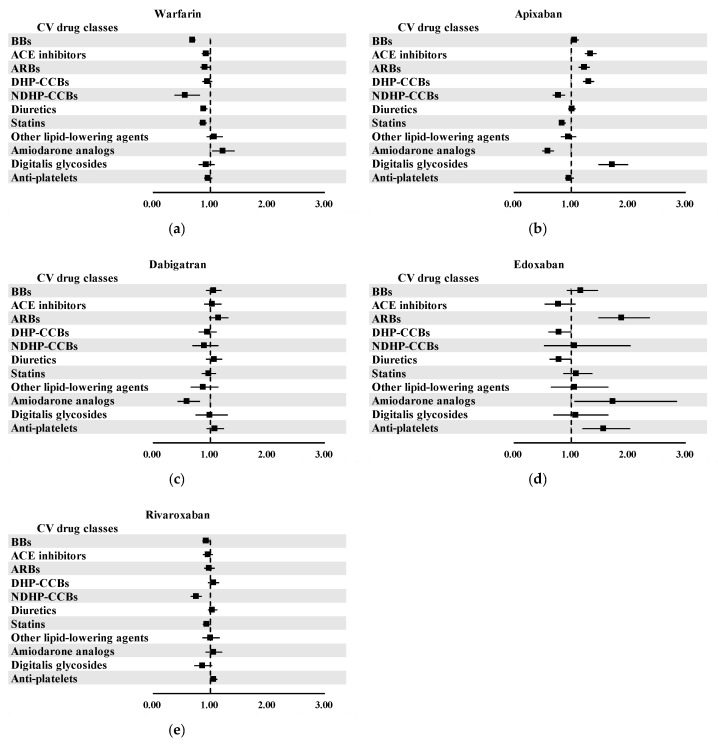
Results of pooled analysis for potential drug interactions between OACs and CV drugs: (**a**) warfarin; (**b**) apixaban; (**c**) dabigatran; (**d**) edoxaban; and (**e**) rivaroxaban.

In the pooled analyses, several classes of CV drugs demonstrated statistically significant associations with an increased risk of UGI AEs when used concomitantly with specific OACs (Figure 3). A significant signal was observed for warfarin when co-administered with amiodarone analogs (aROR, 1.22; 95% CI, 1.04–1.43), though no other CV drug classes showed associations with warfarin. Apixaban showed several significant signals across a range of CV drug classes (Figure 3b). Increased UGI AE risks were observed in apixaban-related ICSRs in combination with ACE inhibitors (aROR, 1.34; 95% CI, 1.24–1.45), ARBs (aROR, 1.23; 95% CI, 1.14–1.33), DHP-CCBs (aROR, 1.30; 95% CI, 1.21–1.41), or digitalis glycosides (aROR, 1.72; 95% CI, 1.49–2.00). Additionally, as shown in Figure 3d, edoxaban was significantly associated with elevated UGI AE risk when co-administered with ARBs (aROR, 1.88; 95% CI, 1.48–2.37), amiodarone analogs (aROR, 1.73; 95% CI, 1.06–2.85), and anti-platelets (aROR, 1.56; 95% CI, 1.20–2.03). These findings suggested potential vulnerabilities when edoxaban is administered concurrently with these drug classes.

**Figure 4:**
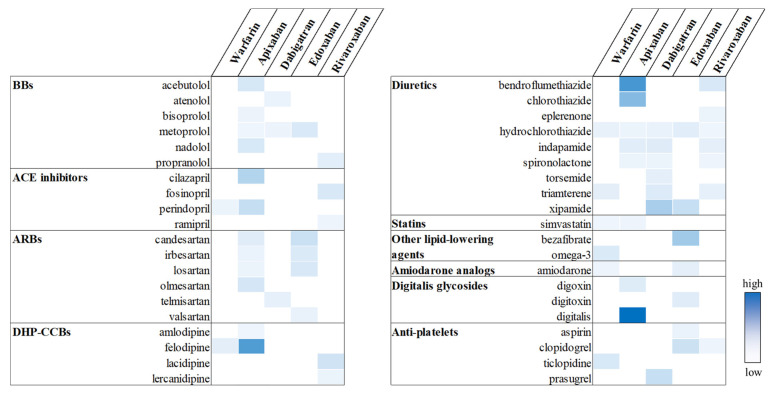
Potential drug interactions between OACs and CV drugs. Statistically significant signals are highlighted in color, with greater intensity indicating higher aROR values.

In the individual drug-level analyses, a total of 62 OAC-CV drug pairs showed statistically significant associations with UGI AEs: 8 for warfarin, 20 for apixaban, 10 for dabigatran, 12 for edoxaban, and 12 for rivaroxaban (Figure 4). Among these, the strongest signals were observed for apixaban–digitalis (aROR, 16.05; 95% CI, 3.94–61.20), apixaban–bendroflumethiazide (aROR, 11.63; 95% CI, 8.97–15.08), and apixaban–felodipine (aROR, 11.10; 95% CI, 8.91–13.81), suggesting a markedly elevated UGI AE risk for these specific combinations. A complete set of results, including both significant and non-significant OAC-CV drug pairs, is provided in Supplementary Tables S1–S5.

## 3. Discussion

This exploratory pharmacovigilance study aimed to update the safety profile of OACs by evaluating their association with UGI AEs, using ICSRs from the FAERS database. Although only dabigatran exhibited a positive signal, suggesting an increased risk of UGI AEs in the disproportionality analysis, subsequent analysis of potential drug interactions with co-medications suggested that not only dabigatran but also other OACs require caution in clinical practice due to possible UGI complications. In particular, apixaban was identified as the OAC most frequently co-reported with CV drugs that have potential drug interactions, suggesting the need for careful consideration when prescribing apixaban and other CV drugs concomitantly.

Approximately 280,000 ICSRs reported OACs as the primary suspected drug in dataset Ⅰ, among which only 3.2% involved UGI AEs. This low reporting rate was attributed to reporting bias, whereby the reporting rate of AEs varies depending on their clinical significance, severity, and awareness [19]. As UGI AEs are often not considered clinically serious or life-threatening, they are often underreported compared to more severe AEs, such as major bleeding [20]. Indeed, despite the low reporting rate of UGI AEs, disproportionality analysis found a statistically significant signal for dabigatran, indicating a potential association with increased risk of UGI AEs. This finding is consistent with a previous study that suggested dabigatran is associated with a higher incidence of UGI AEs compared to warfarin [7].

Although no positive signals were observed for other OACs, investigating the incidence of UGI AEs remains essential for improving patient adherence and preventing potential complications [11]. UGI symptoms, including ulcer-like symptoms, acid reflux, and dyspepsia, are recognized risk factors for UGI bleeding [21]. This emphasizes the necessity of rigorously evaluating GI tolerability in patients prescribed OACs. Furthermore, even when the incidence of UGI AEs associated with a specific OAC is low, the risk could be amplified by concomitant use of interacting drugs [22,23]. This highlights the need for a comprehensive understanding of OAC-related UGI AEs in light of the potential drug interactions.

In the clinical setting, patients prescribed OACs are commonly exposed to polypharmacy rather than exclusive treatment with OACs. This is largely attributable to the high prevalence of comorbid CV disorders among OAC users, which often require concomitant use of CV drugs [15,16]. Given this clinical practice, the present study incorporated 11 CV drug classes as frequently co-administered medications in patients receiving OAC therapy, as well as CYP and P-gp inhibitors, which are the most important drugs evaluated for drug interaction. Previous drug interaction studies using ICSRs have employed a metric approach, wherein two drugs of interest are categorized into four classes of exposure and then evaluated for the occurrence of AEs [24,25,26]. While the metric approach is effective for assessing whether the interaction of two drugs is additive, synergistic, or antagonistic, it has limitations when it comes to considering the effects of other drugs. Therefore, in this study, a multivariate logistic regression analysis was employed to provide a more comprehensive reflection of the effects of CV drugs frequently used by patients taking OACs [27,28].

Given that NSAIDs are well documented to cause UGI AEs such as gastric ulcers and heartburn, they were used as a positive control in the drug interaction analysis for OACs [29]. A potential increase in the UGI AE risk was observed for four of the five OACs, excluding dabigatran. The absence of a signal for dabigatran is explained by the heightened clinical awareness of its UGI risk profile, which has been extensively documented since the RE-LY trial in 2009 [7]. These risks were recognized early after the approval of dabigatran, and clinical guidelines have since recommended the concomitant use of acid suppressants and the avoidance of NSAIDs to mitigate GI bleeding risk [30,31]. As our study included ICSRs from July 2014, it is plausible that these precautions were already being implemented, resulting in more selective NSAID use in patients treated with dabigatran—possibly those at lower risk of UGI AEs. Conversely, the UGI risk profiles of other OACs were not well established at the time of approval. Even now, these risks remain insufficiently characterized or have recently been identified through post-marketing surveillance [8]. The differences in the available GI risk profiles across OACs have influenced prescribing behaviors, particularly the more conservative administration of NSAIDs in dabigatran users. It may have resulted in selection bias and attenuated the observed signal. Additionally, underreporting in spontaneous reporting systems may have contributed to the absence of a detected interaction.

Drug-induced UGI symptoms, including abdominal pain, gastric discomfort, and dyspepsia, are commonly associated with many drugs used in clinical practice. While a variety of medications are implicated in UGI AEs, the underlying mechanisms have been well characterized only for a few drug classes, notably NSAIDs and anticholinergics. For most other drugs, the mechanisms remain largely unexplored [32,33]. Among the OACs, only dabigatran was studied for its association with UGI AEs, which are attributed to the tartaric acid in its formulation rather than dabigatran itself [34,35]. Although UGI AEs are generally less severe than other AEs such as bleeding, they can affect patient adherence. Hwang et al. reported that UGI AEs were the most common reason for discontinuation of dabigatran [36]. Current clinical guidelines from the American College of Cardiology/American Heart Association, the European Society of Cardiology, the American Society of Hematology, and the American College of Chest Physicians recommend OAC therapy ranging from several months to lifelong use. Given this, managing UGI tolerability issues is essential for achieving long-term treatment success. However, these guidelines address drug interactions and polypharmacy primarily from the perspective of reducing bleeding risk and do not provide specific recommendations regarding UGI AEs [5,6,37,38].

To address this gap, the present study focused on UGI tolerability under polypharmacy conditions involving OAC-CV drug combinations and identified several potential interactions. In particular, a high frequency of positive signals was observed for apixaban when co-administered with CV drugs. Apixaban has been perceived as safer than other OACs in vulnerable patients with an elevated bleeding risk, such as those with reduced renal function, advanced age, and low body weight, compared with other OACs [39,40,41]. This perception has led to its higher frequency of use in clinical practice [42]. The positive signals observed for UGI AEs may have been attributable to the intensive use of apixaban in these vulnerable patients. Given this pattern of use, particular attention should be paid to the potential risk of UGI AEs in these high-risk populations. In this context, caution is warranted when interpreting the strong signal observed with the concomitant use of apixaban and digitalis glycosides. Digitalis glycosides are well established to be associated with gastrointestinal toxicity [43], supporting the possibility of a true pharmacologic interaction. At the same time, the absence of a similar signal with other DOACs indicates that residual confounding related to patient characteristics and prescribing preferences may also have contributed. Moreover, combinations involving diuretics–OACs and ARBs–OACs were frequently associated with positive signals, suggesting a broader scope of potential drug-related UGI risks. These findings emphasize the need to evaluate UGI AEs at both the individual OAC and co-administered CV drug levels, thereby providing a complementary perspective to current guidance regarding UGI tolerability.

Several limitations are present in this study. Firstly, the FAERS data have inherent limitations such as underreporting, reporting bias, and the absence of denominator data, which could influence the results and warrant caution in their interpretation. Although these factors affect both disproportionality and drug interaction analyses, certain biases—such as those arising from market share differences—are less likely to affect the drug interaction analysis because comparisons were restricted within each OAC user group, using those without the specific CV drug as the reference. Secondly, the ICSR data from the FAERS is incomplete. The omission of non-suspect drugs frequently occurred, resulting in the underreporting of co-medications. In addition, the incorporation of other important clinical information, such as indications and dosage, often rendered the data inadequate for reliable assessment. To mitigate such incompleteness, ICSRs with missing data on age and sex were excluded, and the number of co-medications was incorporated as a matching variable to adjust for the overall medication burden. Thirdly, this study evaluated signals through a comparison of OAC monotherapy and OACs co-administered with CV drugs; however, there was no identification of the UGI AE risk associated with CV drugs alone. The potential interactions between OACs and non-CV drugs have been insufficiently accounted for in this analysis. An additional important limitation of the present study is that it included only a limited number of CV drugs. In real-world clinical settings, patients are frequently prescribed a broader range of pharmacotherapies.

Despite these limitations, the present study has several methodological strengths that enhance the validity and applicability of the findings. First, we established a clinically relevant definition of UGI AEs by screening approximately 2400 lowest-level terms (LLTs), as the identification of UGI AEs was not standardized in the FAERS database. This rigorous selection process ensured a comprehensive and systematic approach to defining UGI AEs and improved the consistency and clinical interpretability of the definition. Second, a total of 195 drugs, spanning 11 CV drug classes and pharmacokinetic modulators, were included in the present study. This extensive inclusion reflects real-world prescribing patterns, where patients on OAC therapy are commonly exposed to these drugs. By encompassing a broad and clinically significant range of co-medications, the study improves the generalizability and external applicability of its results to clinical practice. Third, independently matched datasets were constructed for each drug of interest to minimize the confounding effects of co-medications and to allow for more precise attribution of the UGI AE risk to specific OAC-CV drug pairs. Such class-specific matching enhances the internal validity of the analysis, facilitating a more focused interpretation of drug interactions in clinical decision making. Several combinations of OACs with commonly used CV drugs, such as ACE inhibitors, ARBs, DHP-CCBs, digitalis glycosides, and amiodarone analogs, were associated with statistically significant signals for UGI AEs. These findings highlight the need for caution when prescribing OACs alongside such medications and support the development of tailored OAC strategies based on co-medication profiles to ensure UGI safety.

## 4. Materials and Methods

### 4.1. Data Resource and Dataset Construction

We analyzed ICSRs available through the FAERS database from 1 July 2014 to 31 December 2023. The FAERS database was composed of seven structured ASCII files, which were merged for analysis. To address duplicate reporting for the same ICSR, records were deduplicated based on the unique ICSR identifier, age, sex, active pharmaceutical ingredient of the product, adverse drug event, indication, reporting date, and reporting country. Only the most recent report per ICSR was retained to ensure data consistency.

Two distinct datasets were then constructed for analysis. Dataset I was created by selecting records in which the drug was reported as the primary suspect and used for traditional disproportionality analysis. The other dataset, dataset II, was established by extracting ICSRs mentioning warfarin, apixaban, dabigatran, edoxaban, or rivaroxaban from the deduplicated latest report dataset, regardless of their role in the report. ICSRs involving more than one OAC or lacking definitive information on age or sex were subsequently excluded. Dataset II was used to evaluate potential drug interactions of OACs subsequently divided into five sub-datasets, each corresponding to one of the OACs.

### 4.2. Definition of Upper Gastrointestinal Adverse Events

UGI AEs of interest were defined using the LLTs from the Medical Dictionary for Regulatory Activities (MedDRA) version 26.0 (Maintenance and Support Organization, McLean, VA, USA). We reviewed 2384 LLTs included in the following two level 1 Standardized MedDRA Queries (SMQs): GI perforation, ulceration, hemorrhage, or obstruction and GI nonspecific inflammation and dysfunctional conditions. To refine the selection toward UGI-specific events, LLTs anatomically related to the oral cavity, esophagus, stomach, and duodenum were included, while those associated with the jejunum, ileum, cecum, colon, sigmoid, rectum, and anus were excluded. With respect to the clinical symptoms, the focus was on LLTs indicative of UGI discomfort, dyspepsia, reflux, ulcers, and inflammation. Consequently, 545 LLTs were identified as UGI AEs of interest (Supplementary Table S6).

### 4.3. Disproportionality Analysis

The conventional disproportionality analysis was conducted by creating a two-by-two contingency table: A, number of ICSRs exposed to an OAC and experiencing UGI AEs; B, number of ICSRs exposed to an OAC but not experiencing UGI AEs; C, number of ICSRs not exposed to an OAC and experiencing UGI AEs; and D, number of ICSRs not exposed to an OAC and not experiencing UGI AEs [44]. Subsequently, the association between OACs and UGI AEs was determined using four metrics: ROR, PRR, IC, and EBGM. The formulas for these metrics are shown in Table 4.

### 4.4. Definition of Drugs of Interest

#### 4.4.1. Positive Control

To reinforce the methodological rigor of the analysis, NSAIDs were incorporated as positive controls, given their well-established association with UGI AEs [45]. The NSAIDs included in this study are listed in Table 5, along with other drugs of interest.

#### 4.4.2. Pharmacokinetic Modulators

Drugs that could pharmacokinetically influence the plasma concentration of OACs were identified as drugs of interest. These included CYP and P-gp inhibitors, which increase systemic exposure to OACs and thereby elevate the risk of UGI AEs. The definition of CYP and P-gp inhibitors was based on the US FDA categorization of modulators as either moderate or strong [46]. Additionally, diltiazem and digoxin, which were not included in the FDA classification, were defined as P-gp inhibitors based on their well-known P-gp inhibiting properties [47].

CYP inducers were excluded from the evaluation of direct effects in the drug interaction analysis. This was due to the fact that risk-reducing effects could not be assessed using ICSR data, which includes only cases reporting AEs. However, we defined CYP inducers according to the US FDA’s definition of moderate or strong inducers in order to adjust for their potential effects as covariates in the drug interaction analysis. The drug list of CYP inhibitors, P-gp inhibitors, and CYP inducers is provided in Table 5.

#### 4.4.3. Cardiovascular Drugs

CV drugs are among the most commonly prescribed co-medications in patients receiving OAC therapy. To assess potential drug interactions of clinical relevance, eleven classes of CV drugs were defined as drugs of interest: BBs, ACE inhibitors, ARBs, DHP-CCBs, NDHP-CCBs, diuretics, statins, other lipid-lowering agents, amiodarone analogs, digitalis glycosides, and anti-platelets. A list of drugs included in each class is presented in Table 5.

#### 4.4.4. Acid-Suppressive Agents

Acid-suppressive agents, which affect the occurrence of UGI AEs, were included as covariates in this study. These agents included PPIs, histamine 2 receptor antagonists (H2RAs), and potassium-competitive acid blockers (PCABs). The included drugs in each class are listed in Table 5.

### 4.5. Drug Interaction Analysis

#### 4.5.1. Classification of OAC-Related ICSRs

The drug interaction analysis was conducted using dataset Ⅱ, which included only ICSRS related to OACs. The classification of these ICSRs was based on two criteria: exposure to drugs of interest as co-medication and experience of UGI AEs. The OAC-related ICSRs were then categorized into four groups: N_00_, no exposure to drugs of interest and no experience of UGI AEs; N_01_, no exposure to drugs of interest and experience of UGI AEs; N_10_, exposure to drugs of interest and no experience of UGI AEs; and N_11_, exposure to drugs of interest and experience of UGI AEs. The initial classification was conducted based on pharmacologic drug classes (e.g., BBs, ACE inhibitors, ARBs), CYP inhibitors, and P-gp inhibitors to enable class-level comparison in the pooled analysis. For individual drug-level analysis, N_10_ and N_11_ were redefined for each specific drug (e.g., atenolol, bisoprolol, metoprolol), while N_00_ and N_01_ remained fixed as ICSRs unexposed to any drug within the corresponding drug class.

#### 4.5.2. Confounding Factors and Matching

In order to address confounding factors associated with the occurrence of UGI AEs, the following variables were included in the analysis: age, sex, acid-suppressive agents, and the number of co-medications. Co-medications were defined as all concurrently reported drugs within a single ICSR. The number of co-medications was stratified into four categories: 1 (only OAC), 2–4, 5–9, and ≥10. Utilizing these confounding factors as covariates, exact matching was conducted for each of the 14 drug classes of interest—including NSAIDs, CYP inhibitors, P-gp inhibitors, and 11 CV drug classes. This process was applied to the five sub-datasets within dataset II to minimize bias by excluding unmatched outliers while preserving the heterogeneity of real-world populations.

#### 4.5.3. Logistic Regression

To estimate the association between co-medications and the risk of UGI AEs in patients receiving OACs, we employed logistic regression analyses. A univariate logistic regression was performed to evaluate the effect of each co-medication on the risk of UGI AEs, estimating cRORs, which were consistent with those derived from the ROR formula described in Table 4. Subsequently, a multivariate logistic regression analysis was conducted to obtain aRORs, incorporating potential confounding factors. These included age, sex, the number of co-medications, acid-suppressive agents, and the concomitant use of NSAIDs, CYP inhibitors, P-gp inhibitors, CYP inducers, and CV drugs.

The estimation of both cRORs and aRORs was conducted only when both N_10_ and N_11_ were ≥3 to ensure statistical reliability and minimize bias from sparse data. All analyses were performed with R software version 4.3.1 (R Foundation for Statistical Computing, Vienna, Austria).

## 5. Conclusions

This exploratory study evaluated potential drug interactions between OACs and commonly co-prescribed CV drugs, focusing on their association with UGI AEs. In the pooled analysis, apixaban showed positive drug interaction signals with ACE inhibitors, ARBs, DHP-CCBs, and digitalis glycosides. At the individual drug level, apixaban was associated with a broader range of CV drugs showing potential interactions compared to other OACs. Our findings highlight the importance of recognizing drug-specific interaction profiles, particularly in the context of polypharmacy with CV drugs, to ensure safe and personalized OAC therapy in clinical practice. For high-risk patients receiving OAC-CV drug combinations, clinicians should closely monitor UGI symptoms and consider the concomitant use of GI protective agents to mitigate potential risks. These considerations help minimize the risk of reduced adherence and treatment discontinuation owing to UGI AEs, ultimately improving the patient outcomes of OACs. Further large-scale cohort studies are warranted to validate these observations and inform clinical decision making.

## Figures and Tables

**Table 1:** Disproportionality analysis for OAC-related UGI AEs.

OAC Type	A	B	C	D	ROR (95% CI)	PRR (95% CI)	IC_025_	EBGM_05_
Warfarin	938	23,263	514,975	12,366,114	0.97 (0.91–1.03)	0.97 (0.91–1.03)	−0.15	0.92
Apixaban	2775	119,696	513,138	12,269,681	0.55 (0.53–0.58)	0.56 (0.54–0.59)	−0.88	0.55
Dabigatran	1643	33,211	514,270	12,356,166	1.19 (1.13–1.25)	1.18 (1.13–1.24)	0.16	1.13
Edoxaban	269	5852	515,644	12,383,525	1.10 (0.98–1.25)	1.10 (0.98–1.24)	−0.07	0.99
Rivaroxaban	3447	92,287	512,466	12,297,090	0.90 (0.87–0.93)	0.90 (0.87–0.93)	−0.21	0.88

A: number of ICSRs exposed to OAC and experiencing UGI AEs; B: number of ICSRs exposed to OAC but not experiencing UGI AEs; C: number of ICSRs not exposed to OAC and experiencing UGI AEs; D: number of ICSRs not exposed to OAC and not experiencing UGI AEs; ROR: reporting odds ratio; PRR: proportional reporting ratio; IC: information component; EBGM: empirical Bayesian geometric mean.

**Table 2:** Demographic characteristics and co-medication information of the OAC-related ICSRs.

Variables		Warfarin, %	Apixaban, %	Dabigatran, %	Edoxaban, %	Rivaroxaban, %
		(n = 86,406)	(n = 126,824)	(n = 30,157)	(n = 7248)	(n = 113,409)
Age group ^†^	0–19	1.62	0.18	0.20	0.14	0.38
	20–39	5.45	2.06	1.33	1.28	3.68
	40–64	29.60	18.73	15.32	14.75	26.24
	65–74	26.58	26.86	28.35	25.62	29.28
	≥75	36.75	52.18	54.81	58.21	40.41
Sex ^†^	Male	46.33	48.56	55.08	52.58	51.08
	Female	53.67	51.44	44.92	47.42	48.92
Number of co-medications ^†^	1 *	7.42	33.57	31.52	10.44	27.93
	2–4	32.59	19.10	20.17	19.95	30.87
	5–9	29.39	23.15	26.67	35.64	21.88
	≥10	30.60	24.19	21.64	33.97	19.32
Co-medications ^†^	BBs	30.03	28.75	35.39	41.39	24.21
	ACE inhibitors	14.03	10.72	16.63	12.93	11.24
	ARBs	11.14	11.95	14.01	22.60	10.09
	DHP-CCBs	10.44	10.05	11.15	19.45	8.61
	NDHP-CCBs	5.66	4.42	6.15	3.31	3.87
	Diuretics	30.04	22.91	27.62	37.40	18.67
	Statins	24.18	22.23	26.54	27.29	19.06
	Other lipid-lowering agents	3.89	2.80	3.54	3.39	2.81
	Amiodarone analogs	5.21	5.62	7.81	6.06	4.70
	Digitalis glycosides	6.98	3.41	7.04	5.52	3.31
	Anti-platelets	17.01	12.69	16.86	13.89	27.85
	CYP inhibitors	23.33	18.90	21.46	21.30	18.79
	CYP inducers	6.71	1.54	1.66	2.26	1.43
	P-gp inhibitors	18.82	13.75	20.32	15.31	12.05
	NSAIDs	5.35	3.90	4.68	7.71	5.48
Acid-suppressive therapy ^†^	All types	25.44	21.41	22.19	39.69	18.67
	PPIs	22.52	19.48	19.92	34.52	17.03
	H2RAs	4.22	2.64	2.83	3.01	2.32
	PCABs	0.16	0.16	0.30	3.46	0.10
Report year	2014 (Q3–Q4)	7.05	0.92	10.97	0.00	2.67
	2015	18.58	2.62	10.67	1.37	11.46
	2016	14.21	4.54	10.55	2.22	15.06
	2017	12.38	6.40	12.63	3.52	12.32
	2018	12.46	8.54	14.67	5.66	12.60
	2019	11.11	11.10	14.40	7.92	9.26
	2020	9.04	12.19	9.66	13.78	19.02
	2021	6.40	18.44	7.10	19.61	6.16
	2022	4.63	17.80	5.59	22.41	5.72
	2023	4.12	17.45	3.76	23.52	5.75
Report country	North America	70.00	68.09	44.74	7.08	70.10
	Europe	21.62	24.47	37.28	54.33	22.23
	Asia	6.67	4.61	13.54	37.58	5.13
	South America	0.60	2.06	2.14	1.01	1.56
	Oceania	0.80	0.72	1.98	0.00	0.74
	Africa	0.31	0.05	0.32	0.00	0.23
	Not specified	2.71	0.57	3.54	0.55	0.64
Reporter occupation	Physicians	24.50	22.93	43.69	53.42	24.47
	Pharmacists	13.33	10.75	9.58	21.34	8.03
	Other health-professionals	26.21	17.54	12.50	14.21	19.06
	Consumer	32.16	47.46	33.24	9.98	46.54
	Others	4.38	1.33	0.99	1.05	1.89

* means ICSRs reported with only OACs. ^†^ means variables used for matching.

**Table 3:** Results of drug interaction analysis with NSAIDs.

OAC Type	N_11_	N_10_	N_01_	N_00_	cROR (95% CI)	aROR (95% CI)
Warfarin	496	4123	4821	70,065	1.75 (1.59–1.93)	1.39 (1.25–1.54)
Apixaban	637	4310	4447	74,503	2.48 (2.27–2.71)	1.95 (1.78–2.14)
Dabigatran	105	1306	1145	17,863	1.26 (1.02–1.55)	1.21 (0.97–1.49)
Edoxaban	62	493	354	5358	1.91 (1.43–2.54)	1.77 (1.29–2.40)
Rivaroxaban	695	5517	4455	70,890	2.01 (1.84–2.18)	1.86 (1.71–2.03)

N_00_: no exposure to NSAIDs and no experience of UGI AEs; N_01_: no exposure to NSAIDs and experience of UGI AEs; N_10_: exposure to NSAIDs and no experience of UGI AEs; N_11_: exposure to NSAIDs and experience of UGI AEs; OAC: oral anticoagulant; cROR: crude reporting odds ratio; aROR: adjusted reporting odds ratio; CI: confidence interval.

**Table 4:** Formulas for disproportionality analysis metrics.

Metrics	Formulas	Threshold
ROR	$ROR=\frac{A/B}{C/D}$	95% LCI > 1 and A ≥ 3
	$95\%CI=e^{\ln\left(ROR\right)\pm 1.96\sqrt{\frac{1}{A}+\frac{1}{B}+\frac{1}{C}+\frac{1}{D}}\;}$	
PRR	$PRR=\frac{A/(A+B)}{C/(C+D)}$	95% LCI > 1 and A ≥ 3
	$95\%CI=e^{\ln\left(PRR\right)\pm 1.96\sqrt{\frac{1}{A}+\frac{1}{B}+\frac{1}{C}+\frac{1}{D}}\;}$	
IC	$IC=\log_{2}\frac{A+0.5}{\frac{\left(A+B\right)\times \left(C+D\right)}{\left(A+B+C+D\right)}+0.5}$	IC_025_ > 0
	${IC}_{025}=IC-3.3\times {\left(A+0.5\right)}^{-0.5}-2\times {\left(A+0.5\right)}^{-1.5}$	
EBGM	$EBGM=\frac{A\times (A+B+C+D)}{\left(A+C)\times (A+B\right)}$	EBGM_05_ > 2
	${EBGM}_{05}=e^{\ln\left(EBGM\right)-1.64\sqrt{\frac{1}{A}+\frac{1}{B}+\frac{1}{C}+\frac{1}{D}}\;}$	

A: number of ICSRs exposed to OAC and experiencing UGI AEs; B: number of ICSRs exposed to OAC but not experiencing UGI AEs; C: number of ICSRs not exposed to OAC and experiencing UGI AEs; D: number of ICSRs not exposed to OAC and not experiencing UGI AEs; ROR: reporting odds ratio; PRR: proportional reporting ratio; IC: information component; EBGM: empirical Bayesian geometric mean; LCI: lower limit of 95% confidence interval.

**Table 5:** List of drugs of interest.

Classification		Drugs
Positive controls	NSAIDs	Aceclofenac, celecoxib, diclofenac, diflunisal, etodolac, flurbiprofen, ibuprofen, indomethacin, ketoprofen, ketorolac, loxoprofen, mefenamic acid, meloxicam, nabumetone, naproxen, nimesulide, oxaprozin, piroxicam, salicylate, salsalate, sulindac
Pharmacokinetic modulators	CYP inhibitors	Abiraterone, amiodarone, aprepitant, bupropion, cenobamate, ceritinib, cinacalcet, ciprofloxacin, clarithromycin, clopidogrel, cobicistat, conivaptan, crizotinib, dasabuvir, deferasirox, diltiazem, dronedarone, duloxetine, elvitegravir, erythromycin, felbamate, fluconazole, fluoxetine, fluvoxamine, gemfibrozil, idelalisib, imatinib, indinavir, isavuconazole, itraconazole, ketoconazole, lopinavir, lorcaserin, methoxsalen, mexiletine, miconazole, mirabegron, nefazodone, nelfinavir, ombitasvir, paritaprevir, paroxetine, piperine, posaconazole, quinidine, ritonavir, rolapitant, saquinavir, telithromycin, terbinafine, teriflunomide, ticlopidine, tipranavir, vemurafenib, verapamil, voriconazole
	P-gp inhibitors	Amiodarone, clarithromycin, cobicistat, cyclosporine, digoxin, diltiazem, dronedarone, erythromycin, itraconazole, ketoconazole, lapatinib, lopinavir, propafenone, quinidine, ranolazine, ritonavir, saquinavir, sofosbuvir, velpatasvir, verapamil, voxilaprevir
	CYP inducers	Apalutamide, bosentan, carbamazepine, cenobamate, dabrafenib, efavirenz, enzalutamide, etravirine, ivacaftor, ivosidenib, lorlatinib, lumacaftor, mitotane, pexidartinib, phenobarbital, phenytoin, primidone, rifampin (rifampicin), sotorasib, teriflunomide
Cardiovascular drugs	BBs	Acebutolol, alprenolol, arotinolol, atenolol, betzxolol, bevantolol, bisoprolol, carteolol, carvedilol, celiprolol, esmolol, labetalol, landiolol, levobunolol, metoprolol, moprolol, nadolol, nebivolol, nipradilol, penbutolol, pindolol, propranolol, sotalol, stanozolol, talinolol, timolol
	ACE inhibitors	Alacepril, benazepril, captopril, cilazapril, delapril, enalapril, fosinopril, imidapril, lisinopril, moexipril, pentopril, perindopril, quinapril, ramipril, temocapril, trandolapril, zofenopril
	ARBs	Azilsartan, candesartan, eprosartan, fimasartan, irbesartan, losartan, olmesartan, tasosartan, telmisartan, valsartan
	DHP-CCBs	Amlodipine, azelnidipine, barnidipine, benidipine, cilnidipine, clevidipine, felodipine, isradipine, lacidipine, lercanidipine, levamlopdipine, manidipine, nicardipine, nifedipine, nilvadipine, nimodipine, nisoldipine, nitrendipine
	NDHP-CCBs	Diltiazem, verapamil
	Diuretics	Althiazide, amiloride, azosemide, bendroflumethiazide, benzthiazide, buthiazide, chlorothiazide, chlorthalidone, clopamide, cyclothiazide, eplerenone, ethacrynic acid, flumethiazide, furosemide, hydrochlorothiazide, hydroflumethiazide, indapamide, methylclothiazide, metolazone, sprionolacton, torsemide, triamterene, trichlormethiazide, xipamide
	Statins	Atorvastatin, cerivastatin, fluvastatin, lovastatin, pentostatin, pitavastatin, pravastatin, rosuvastatin, simvastatin
	Other lipid-lowering agents	Alirocumab, bezafibrate, ciprofibrate, clofibrate, evolocumab, ezetimibe, fenofibrate, gemfibrozil, omega-3, pemafibrate
	Amiodarone analogs	Amiodaorone, dronedarone
	Digitalis glycosides	Digoxin, digitoxin, digitalis
	Anti- platelets	Abciximab, anagrelide, aspirin, cangrelor, cilostazol, clopidogrel, dipyridamole, eptifibatide, indobufen, ozagrel, prasugrel, sarpogrelate, sulodexide, ticagrelor, ticlopidine, tirofiban, triflusal
Acid-suppressive agents	PPIs	Dexlansoprazole, esomeprazole, ilaprazole, lansoprazole, omeprazole, pantoprazole, rabeprazole
	H2RAs	Cimetidine, famotidine, lafutidine, nizatidine, ranitidine, roxatidine
	PCABs	Tegoprazan, vonoprazan

NSAIDs: non-steroidal anti-inflammatory drugs; CYP: cytochrome P450 enzyme; P-gp: P-glycoprotein; BB: beta-adrenergic receptor blocker; ACE: angiotensin-converting enzyme; ARB: angiotensin receptor blocker; DHP: dihydropyridine; NDHP: non-dihydropyridine; CCB: calcium channel blocker; PPI: proton pump inhibitor; H2RA: histamine 2 receptor antagonists; PCAB: potassium-competitive acid blocker.

## Data Availability

The FDA Adverse Event Reporting System (FAERS) quarterly data extract files used in this study are publicly available at the following link: https://www.fda.gov/drugs/fdas-adverse-event-reporting-system-faers/fda-adverse-event-reporting-system-faers-latest-quarterly-data-files (accessed on 3 April 2024).

## Supplementary Materials

The following supporting information can be downloaded at https://www.mdpi.com/article/10.3390/ph18091311/s1: Supplementary Table S1: Potential drug interactions between warfarin and drugs of interest. Supplementary Table S2: Potential drug interactions between apixaban and drugs of interest. Supplementary Table S3: Potential drug interactions between dabigatran and drugs of interest. Supplementary Table S4: Potential drug interactions between edoxaban and drugs of interest. Supplementary Table S5: Potential drug interactions between rivaroxaban and drugs of interest. Supplementary Table S6: Definition of upper gastrointestinal adverse events.

## Abbreviations

The following abbreviations are used in this manuscript:

ACE	Angiotensin-converting enzyme
AE	Adverse event
ARB	Angiotensin receptor blocker
aROR	Adjusted reporting odds ratio
BB	Beta-adrenergic receptor blocker
CI	Confidence interval
cROR	Crude reporting odds ratio
CV	Cardiovascular
CYP	Cytochrome P450 enzyme
DHP-CCB	Dihydropyridine calcium channel blocker
DOAC	Direct oral anticoagulant
EBGM	Empirical Bayesian geometric mean
FAERS	Food and Drug Administration Adverse Event Reporting System
GERD	Gastroesophageal reflux disease
GI	Gastrointestinal
H2RA	Histamine 2 receptor antagonist
IC	Information component
ICSR	Individual case safety report
LCI	Lower limit of 95% confidence interval
LLT	Lowest-level term
NDHP-CCB	Non-dihydropyridine calcium channel blocker
NSAID	Non-steroidal anti-inflammatory drug
OAC	Oral anticoagulant
PCAB	Potassium-competitive acid blocker
P-gp	P-glycoprotein
PPI	Proton pump inhibitor
PRR	Proportional reporting ratio
ROR	Reporting odds ratio
