# Supplementary material for: Analysis of Upper Gastrointestinal Adverse Events Associated with Oral Anticoagulants and Potential Drug Interactions with Cardiovascular Drugs: Exploratory Study Using FDA Adverse Event Reporting System
Source: Pharmaceuticals (Basel). 2025 Sep 1;18(9):1311. doi: 10.3390/ph18091311 (PMC12472349; doi:10.3390/ph18091311)
Supplement: Supplementary file 1 [file pharmaceuticals-18-01311-s001.zip › pharmaceuticals-3821144-supplementary.pdf]

Supplementary Materials

Supplementary Table S1. Potential drug interactions between warfarin and drugs of interest.

Drugs of interest	N_{11}	N_{10}	N_{01}	N_{00}	Crude ROR (95% CI)	Adjusted ROR (95% CI)
**CYP inhibitors**	**1,646**	**18,513**	**3,682**	**56,003**	**1.35 (1.27–1.44)**	**1.19 (1.11–1.27)**
abiraterone	7	285	3,682	56,003	0.37 (0.18–0.79)	0.44 (0.19–0.86)
amiodarone	349	3,875	3,682	56,003	1.37 (1.22–1.54)	1.31 (1.06–1.61)
aprepitant	6	92	3,682	56,003	0.99 (0.43–2.27)	0.74 (0.29–1.57)
bupropion	63	853	3,682	56,003	1.12 (0.87–1.45)	0.90 (0.68–1.16)
cenobamate	0	0	3,682	56,003	NA	NA
ceritinib	0	9	3,682	56,003	NA	NA
cinacalcet	23	378	3,682	56,003	0.93 (0.61–1.41)	0.75 (0.48–1.13)
ciprofloxacin	113	1,127	3,682	56,003	1.53 (1.25–1.86)	1.20 (0.97–1.47)
clarithromycin	68	532	3,682	56,003	1.94 (1.51–2.51)	1.76 (1.28–2.39)
clopidogrel	242	3,403	3,682	56,003	1.08 (0.95–1.24)	1.08 (0.92–1.27)
cobicistat	2	55	3,682	56,003	NA	NA
conivaptan	0	0	3,682	56,003	NA	NA
crizotinib	6	68	3,682	56,003	1.34 (0.58–3.09)	1.16 (0.45–2.47)
dasabuvir	11	54	3,682	56,003	3.10 (1.62–5.93)	3.04 (1.46–5.85)
deferasirox	10	81	3,682	56,003	1.88 (0.97–3.63)	1.62 (0.79–3.00)
diltiazem	269	3,667	3,682	56,003	1.12 (0.98–1.27)	1.03 (0.90–1.18)
dronedarone	25	271	3,682	56,003	1.40 (0.93–2.12)	1.64 (1.01–2.56)
duloxetine	334	1,415	3,682	56,003	3.59 (3.17–4.06)	2.27 (1.98–2.61)
elvitegravir	2	24	3,682	56,003	NA	NA
erythromycin	13	169	3,682	56,003	1.17 (0.66–2.06)	1.00 (0.52–1.78)
felbamate	0	0	3,682	56,003	NA	NA
fluconazole	46	743	3,682	56,003	0.94 (0.70–1.27)	0.72 (0.52–0.96)
fluoxetine	86	997	3,682	56,003	1.31 (1.05–1.64)	1.12 (0.89–1.40)
fluvoxamine	6	29	3,682	56,003	3.15 (1.31–7.58)	2.47 (0.91–5.62)
gemfibrozil	17	210	3,682	56,003	1.23 (0.75–2.02)	1.46 (0.82–2.45)
idelalisib	8	73	3,682	56,003	1.67 (0.80–3.46)	1.75 (0.78–3.43)
imatinib	11	116	3,682	56,003	1.44 (0.78–2.68)	1.43 (0.72–2.55)
indinavir	0	9	3,682	56,003	NA	NA
isavuconazole	0	1	3,682	56,003	NA	NA
itraconazole	3	69	3,682	56,003	0.66 (0.21–2.10)	0.60 (0.14–1.66)
ketoconazole	12	192	3,682	56,003	0.95 (0.53–1.70)	0.80 (0.41–1.45)
lopinavir	1	64	3,682	56,003	NA	NA
lorcaserin	1	25	3,682	56,003	NA	NA
methoxsalen	0	1	3,682	56,003	NA	NA
mexiletine	2	108	3,682	56,003	NA	NA
miconazole	5	178	3,682	56,003	0.43 (0.18–1.04)	0.39 (0.14–0.87)
mirabegron	18	365	3,682	56,003	0.75 (0.47–1.21)	0.69 (0.41–1.07)
nefazodone	2	8	3,682	56,003	NA	NA
nelfinavir	0	9	3,682	56,003	NA	NA
ombitasvir	14	82	3,682	56,003	2.60 (1.47–4.58)	2.55 (1.33–4.57)
paritaprevir	14	81	3,682	56,003	2.63 (1.49–4.64)	2.59 (1.35–4.64)
paroxetine	56	812	3,682	56,003	1.05 (0.80–1.38)	0.89 (0.67–1.17)
piperine	0	0	3,682	56,003	NA	NA
posaconazole	3	41	3,682	56,003	1.11 (0.34–3.60)	0.80 (0.19–2.22)
quinidine	1	36	3,682	56,003	NA	NA
ritonavir	21	345	3,682	56,003	0.93 (0.60–1.44)	0.92 (0.55–1.48)
rolapitant	0	5	3,682	56,003	NA	NA
saquinavir	0	3	3,682	56,003	NA	NA
telithromycin	0	1	3,682	56,003	NA	NA
terbinafine	7	160	3,682	56,003	0.67 (0.31–1.42)	0.58 (0.24–1.15)
teriflunomide	8	97	3,682	56,003	1.25 (0.61–2.58)	1.02 (0.45–2.00)
ticlopidine	11	49	3,682	56,003	3.41 (1.77–6.57)	3.08 (1.50–5.78)
tipranavir	0	3	3,682	56,003	NA	NA
vemurafenib	0	23	3,682	56,003	NA	NA
verapamil	58	913	3,682	56,003	0.97 (0.74–1.26)	0.91 (0.65–1.25)
voriconazole	4	123	3,682	56,003	0.49 (0.18–1.34)	0.45 (0.14–1.07)
**P-gp inhibitors**	**983**	**12,775**	**4,354**	**61,737**	**1.09 (1.02–1.17)**	**1.03 (0.93–1.14)**
amiodarone	349	3,875	4,354	61,737	1.28 (1.14–1.43)	1.23 (1.08–1.40)
clarithromycin	68	532	4,354	61,737	1.81 (1.41–2.34)	1.41 (1.05–1.85)
cobicistat	2	55	4,354	61,737	NA	NA
cyclosporine	45	588	4,354	61,737	1.09 (0.80–1.47)	0.89 (0.64–1.20)
digoxin	362	5,632	4,354	61,737	0.91 (0.82–1.02)	3.13 (0.54–25.46)
diltiazem	269	3,666	4,354	61,737	1.04 (0.92–1.18)	0.91 (0.62–1.29)
dronedarone	25	271	4,354	61,737	1.31 (0.87–1.97)	1.14 (0.72–1.72)
erythromycin	13	169	4,354	61,737	1.09 (0.62–1.92)	0.77 (0.41–1.33)
itraconazole	3	69	4,354	61,737	0.62 (0.19–1.96)	0.46 (0.11–1.26)
ketoconazole	12	192	4,354	61,737	0.89 (0.49–1.59)	0.56 (0.29–0.99)
lapatinib	4	12	4,354	61,737	4.73 (1.52–14.66)	3.92 (1.09–11.38)
lopinavir	1	64	4,354	61,737	NA	NA
propafenone	31	301	4,354	61,737	1.46 (1.01–2.12)	1.44 (0.96–2.06)
quinidine	1	36	4,354	61,737	NA	NA
ranolazine	13	293	4,354	61,737	0.63 (0.36–1.10)	0.59 (0.32–1.01)
ritonavir	21	345	4,354	61,737	0.86 (0.55–1.34)	0.74 (0.46–1.13)
saquinavir	0	3	4,354	61,737	NA	NA
sofosbuvir	5	214	4,354	61,737	0.33 (0.14–0.80)	0.34 (0.12–0.75)
velpatasvir	0	34	4,354	61,737	NA	NA
verapamil	58	913	4,354	61,737	0.90 (0.69–1.18)	0.73 (0.54–0.97)
voxilaprevir	0	1	4,354	61,737	NA	NA
**BBs**	**1,500**	**24,448**	**3,833**	**50,064**	**0.80 (0.75–0.85)**	**0.70 (0.65–0.75)**
acebutolol	5	63	3,833	50,064	1.04 (0.42–2.58)	0.80 (0.28–1.81)
alprenolol	0	0	3,833	50,064	NA	NA
arotinolol	0	9	3,833	50,064	NA	NA
atenolol	110	2,179	3,833	50,064	0.66 (0.54–0.80)	0.58 (0.47–0.71)
betzxolol	0	0	3,833	50,064	NA	NA
bevantolol	0	0	3,833	50,064	NA	NA
bisoprolol	305	6,023	3,833	50,064	0.66 (0.59–0.75)	0.62 (0.55–0.71)
carteolol	0	3	3,833	50,064	NA	NA
carvedilol	298	4,674	3,833	50,064	0.83 (0.74–0.94)	0.74 (0.65–0.85)
celiprolol	4	27	3,833	50,064	1.94 (0.68–5.53)	1.85 (0.54–4.84)
esmolol	0	18	3,833	50,064	NA	NA
labetalol	11	226	3,833	50,064	0.64 (0.35–1.17)	0.47 (0.24–0.84)
landiolol	0	14	3,833	50,064	NA	NA
levobunolol	0	3	3,833	50,064	NA	NA
metoprolol	679	9,481	3,833	50,064	0.94 (0.86–1.02)	0.78 (0.71–0.85)
moprolol	0	0	3,833	50,064	NA	NA
nadolol	2	118	3,833	50,064	NA	NA
nebivolol	34	672	3,833	50,064	0.66 (0.47–0.93)	0.59 (0.41–0.83)
nipradilol	0	0	3,833	50,064	NA	NA
penbutolol	0	2	3,833	50,064	NA	NA
pindolol	0	15	3,833	50,064	NA	NA
propranolol	36	525	3,833	50,064	0.90 (0.64–1.26)	0.73 (0.51–1.01)
sotalol	64	949	3,833	50,064	0.88 (0.68–1.14)	0.82 (0.63–1.06)
stanozolol	0	0	3,833	50,064	NA	NA
talinolol	0	0	3,833	50,064	NA	NA
timolol	22	438	3,833	50,064	0.66 (0.43–1.01)	0.55 (0.35–0.83)
**ACE inhibitors**	**778**	**11,345**	**4,542**	**63,062**	**0.95 (0.88–1.03)**	**0.93 (0.85–1.01)**
alacepril	0	3	4,542	63,062	NA	NA
benazepril	33	410	4,542	63,062	1.12 (0.78–1.60)	0.98 (0.67–1.38)
captopril	7	131	4,542	63,062	0.74 (0.35–1.59)	0.72 (0.30–1.43)
cilazapril	3	14	4,542	63,062	2.98 (0.85–10.36)	2.91 (0.67–9.04)
delapril	0	4	4,542	63,062	NA	NA

enalapril	102	1,402	4,542	63,062	1.01 (0.82–1.24)	1.01 (0.82–1.24)
fosinopril	4	80	4,542	63,062	0.69 (0.25–1.90)	0.68 (0.20–1.64)
imidapril	4	61	4,542	63,062	0.91 (0.33–2.50)	0.85 (0.26–2.10)
lisinopril	379	5,248	4,542	63,062	1.00 (0.90–1.12)	0.93 (0.83–1.04)
moexipril	0	14	4,542	63,062	NA	NA
pentopril	0	0	4,542	63,062	NA	NA
perindopril	84	916	4,542	63,062	1.27 (1.02–1.60)	1.37 (1.08–1.72)
quinapril	11	176	4,542	63,062	0.87 (0.47–1.60)	0.92 (0.47–1.62)
ramipril	168	2,939	4,542	63,062	0.79 (0.68–0.93)	0.84 (0.71–0.99)
temocapril	0	9	4,542	63,062	NA	NA
trandolapril	4	112	4,542	63,062	0.50 (0.18–1.34)	0.45 (0.14–1.07)
zofenopril	0	23	4,542	63,062	NA	NA
**ARBs**	**615**	**9,010**	**4,695**	**65,368**	**0.95 (0.87–1.04)**	**0.91 (0.83–1.00)**
azilsartan	4	83	4,695	65,368	0.67 (0.25–1.83)	0.57 (0.17–1.38)
candesartan	77	999	4,695	65,368	1.07 (0.85–1.36)	1.04 (0.81–1.31)
eprosartan	0	8	4,695	65,368	NA	NA
fimasartan	0	0	4,695	65,368	NA	NA
irbesartan	39	823	4,695	65,368	0.66 (0.48–0.91)	0.63 (0.45–0.86)
losartan	268	3,747	4,695	65,368	1.00 (0.88–1.13)	0.92 (0.81–1.05)
olmesartan	55	632	4,695	65,368	1.21 (0.92–1.60)	1.11 (0.83–1.46)
tasosartan	0	0	4,695	65,368	NA	NA
telmisartan	37	496	4,695	65,368	1.04 (0.74–1.45)	0.88 (0.62–1.23)
valsartan	161	2,477	4,695	65,368	0.90 (0.77–1.06)	0.92 (0.78–1.08)
**DHP-CCBs**	**615**	**8,405**	**4,712**	**65,974**	**1.02 (0.94–1.12)**	**0.95 (0.87–1.04)**
amlodipine	510	6,620	4,712	65,974	1.08 (0.98–1.19)	1.00 (0.90–1.10)
azelnidipine	2	52	4,712	65,974	NA	NA
barnidipine	4	30	4,712	65,974	1.87 (0.66–5.30)	2.37 (0.70–6.07)
benidipine	0	82	4,712	65,974	NA	NA
cilnidipine	1	49	4,712	65,974	NA	NA
clevidipine	0	1	4,712	65,974	NA	NA
felodipine	24	191	4,712	65,974	1.76 (1.15–2.69)	1.85 (1.18–2.80)
isradipine	0	8	4,712	65,974	NA	NA
lacidipine	1	53	4,712	65,974	NA	NA
lercanidipine	11	341	4,712	65,974	0.45 (0.25–0.82)	0.45 (0.23–0.79)
levamlopdipine	0	0	4,712	65,974	NA	NA
manidipine	0	27	4,712	65,974	NA	NA
nicardipine	6	156	4,712	65,974	0.54 (0.24–1.22)	0.43 (0.17–0.89)
nifedipine	67	991	4,712	65,974	0.95 (0.74–1.21)	0.79 (0.61–1.01)
nilvadipine	0	1	4,712	65,974	NA	NA
nimodipine	2	3	4,712	65,974	NA	NA
nisoldipine	2	21	4,712	65,974	NA	NA
nitrendipine	0	12	4,712	65,974	NA	NA
**NDHP-CCBs**	**319**	**4,570**	**4,976**	**69,508**	**0.98 (0.87–1.10)**	**0.56 (0.38–0.82)**
diltiazem	269	3,667	4,976	69,508	1.02 (0.90–1.16)	0.79 (0.68–0.91)
verapamil	58	913	4,976	69,508	0.89 (0.68–1.16)	0.70 (0.52–0.93)
**Diuretics**	**1,774**	**24,170**	**3,563**	**50,391**	**1.04 (0.98–1.10)**	**0.89 (0.83–0.95)**
althiazide	1	6	3,563	50,391	NA	NA
amiloride	14	220	3,563	50,391	0.90 (0.52–1.55)	0.72 (0.40–1.21)
azosemide	26	403	3,563	50,391	0.91 (0.61–1.36)	0.68 (0.44–1.01)
bendroflumethiazide	4	217	3,563	50,391	0.26 (0.10–0.70)	0.26 (0.08–0.61)
benzthiazide	0	0	3,563	50,391	NA	NA
buthiazide	1	2	3,563	50,391	NA	NA
chlorothiazide	3	35	3,563	50,391	1.21 (0.37–3.94)	0.88 (0.21–2.48)
chlorthalidone	19	196	3,563	50,391	1.37 (0.86–2.20)	1.19 (0.71–1.88)
clopamide	0	0	3,563	50,391	NA	NA
cyclothiazide	0	0	3,563	50,391	NA	NA
eplerenone	27	508	3,563	50,391	0.75 (0.51–1.11)	0.74 (0.49–1.08)
ethacrynic acid	1	16	3,563	50,391	NA	NA
flumethiazide	0	0	3,563	50,391	NA	NA
furosemide	1,310	17,589	3,563	50,391	1.05 (0.99–1.12)	0.90 (0.84–0.97)
hydrochlorothiazide	451	3,283	3,563	50,391	1.94 (1.75–2.16)	1.52 (1.35–1.71)
hydroflumethiazide	0	0	3,563	50,391	NA	NA
indapamide	20	305	3,563	50,391	0.93 (0.59–1.46)	0.85 (0.52–1.31)
methylclothiazide	0	0	3,563	50,391	NA	NA
metolazone	77	1,045	3,563	50,391	1.04 (0.82–1.32)	0.80 (0.62–1.01)
spironolacton	398	5,689	3,563	50,391	0.99 (0.89–1.10)	0.79 (0.70–0.89)
torsemide	95	1,650	3,563	50,391	0.81 (0.66–1.00)	0.64 (0.52–0.80)
triamterene	68	342	3,563	50,391	2.81 (2.16–3.66)	1.85 (1.39–2.42)
trichlormethiazide	11	177	3,563	50,391	0.88 (0.48–1.62)	0.63 (0.32–1.11)
xipamide	0	1	3,563	50,391	NA	NA
**Statins**	**1,372**	**19,517**	**3,932**	**54,501**	**0.97 (0.91–1.04)**	**0.87 (0.81–0.94)**
atorvastatin	564	9,418	3,932	54,501	0.83 (0.76–0.91)	0.74 (0.67–0.81)
cerivastatin	0	2	3,932	54,501	NA	NA
fluvastatin	4	96	3,932	54,501	0.58 (0.21–1.57)	0.48 (0.15–1.16)
lovastatin	31	525	3,932	54,501	0.82 (0.57–1.18)	0.74 (0.50–1.05)
pentostatin	0	2	3,932	54,501	NA	NA
pitavastatin	5	173	3,932	54,501	0.40 (0.16–0.98)	0.34 (0.12–0.74)
pravastatin	156	2,153	3,932	54,501	1.00 (0.85–1.19)	0.85 (0.72–1.01)
rosuvastatin	221	2,838	3,932	54,501	1.08 (0.94–1.24)	0.98 (0.84–1.14)
simvastatin	460	5,062	3,932	54,501	1.26 (1.14–1.39)	1.14 (1.02–1.27)
**Other lipid-lowering agents**	**284**	**3,078**	**4,915**	**69,359**	**1.30 (1.15–1.48)**	**1.07 (0.94–1.22)**
alirocumab	4	111	4,915	69,359	0.51 (0.19–1.38)	0.51 (0.16–1.21)
bezafibrate	6	67	4,915	69,359	1.26 (0.55–2.91)	1.00 (0.39–2.14)
ciprofibrate	0	3	4,915	69,359	NA	NA
clofibrate	1	1	4,915	69,359	NA	NA
evolocumab	10	283	4,915	69,359	0.50 (0.27–0.94)	0.52 (0.26–0.92)
ezetimibe	76	1,207	4,915	69,359	0.89 (0.70–1.12)	0.80 (0.63–1.01)
fenofibrate	45	846	4,915	69,359	0.75 (0.56–1.01)	0.64 (0.47–0.86)
gemfibrozil	17	210	4,915	69,359	1.14 (0.70–1.87)	0.89 (0.52–1.42)
linolenic acid	0	1	4,915	69,359	NA	NA
omega-3	146	591	4,915	69,359	3.49 (2.90–4.19)	2.40 (1.97–2.90)
pemafibrate	0	1	4,915	69,359	NA	NA
**Amiodarone analogs**	**374**	**4,128**	**4,920**	**69,996**	**1.29 (1.16–1.44)**	**1.22 (1.04–1.43)**
amiodarone	349	3,875	4,920	69,996	1.28 (1.14–1.44)	1.20 (1.02–1.41)
dronedarone	25	271	4,920	69,996	1.31 (0.87–1.98)	1.34 (0.85–2.02)
**Digitalis glycosides**	**363**	**5,669**	**4,909**	**67,893**	**0.89 (0.79–0.99)**	**0.93 (0.80–1.08)**
digoxin	362	5,632	4,909	67,893	0.89 (0.80–0.99)	0.94 (0.80–1.09)
digitoxin	0	23	4,909	67,893	NA	NA
digitalis	1	19	4,909	67,893	NA	NA
**Anti-platelets**	**1,003**	**13,688**	**4,326**	**60,850**	**1.03 (0.96–1.11)**	**0.96 (0.89–1.04)**
aspirin	853	11,622	4,326	60,850	1.03 (0.96–1.11)	0.98 (0.90–1.06)
clopidogrel	242	3,403	4,326	60,850	1.00 (0.87–1.14)	0.91 (0.78–1.06)
cilostazol	16	197	4,326	60,850	1.14 (0.69–1.90)	1.06 (0.61–1.72)
dipyridamole	9	168	4,326	60,850	0.75 (0.39–1.47)	0.68 (0.32–1.26)
triflusal	0	1	4,326	60,850	NA	NA
ticlopidine	11	49	4,326	60,850	3.16 (1.64–6.08)	2.60 (1.27–4.89)
tirofiban	0	14	4,326	60,850	NA	NA
eptifibatide	0	11	4,326	60,850	NA	NA
abciximab	1	4	4,326	60,850	NA	NA
sulodexide	0	3	4,326	60,850	NA	NA
indobufen	0	0	4,326	60,850	NA	NA
anagrelide	3	33	4,326	60,850	1.28 (0.39–4.17)	1.19 (0.28–3.35)
cangrelor	0	8	4,326	60,850	NA	NA
ozagrel	0	0	4,326	60,850	NA	NA
prasugrel	11	144	4,326	60,850	1.07 (0.58–1.99)	1.08 (0.55–1.92)
sarpogrelate	1	43	4,326	60,850	NA	NA
ticagrelor	17	210	4,326	60,850	1.14 (0.69–1.87)	1.29 (0.76–2.07)

Supplementary Table S2. Potential drug interactions between apixaban and drugs of interest.

Drugs of interest	N_{11}	N_{10}	N_{01}	N_{00}	Crude ROR (95% CI)	Adjusted ROR (95% CI)
**CYP inhibitors**	**1,416**	**22,546**	**3,689**	**56,515**	**0.96 (0.90–1.02)**	**1.00 (0.93–1.08)**
abiraterone	15	372	3,689	56,515	0.62 (0.37–1.04)	0.75 (0.43–1.23)
amiodarone	249	6,365	3,689	56,515	0.60 (0.53–0.68)	0.52 (0.42–0.64)
aprepitant	7	143	3,689	56,515	0.75 (0.35–1.60)	0.60 (0.25–1.20)
bupropion	308	778	3,689	56,515	6.06 (5.29–6.95)	4.38 (3.77–5.08)
cenobamate	0	6	3,689	56,515	NA	NA
ceritinib	1	7	3,689	56,515	NA	NA
cinacalcet	8	119	3,689	56,515	1.03 (0.50–2.11)	0.86 (0.38–1.66)
ciprofloxacin	64	893	3,689	56,515	1.10 (0.85–1.42)	0.80 (0.61–1.04)
clarithromycin	18	310	3,689	56,515	0.89 (0.55–1.43)	0.58 (0.34–0.94)
clopidogrel	275	4,827	3,689	56,515	0.87 (0.77–0.99)	0.84 (0.72–0.98)
cobicistat	4	44	3,689	56,515	1.39 (0.50–3.88)	0.88 (0.25–2.32)
conivaptan	0	0	3,689	56,515	NA	NA
crizotinib	3	68	3,689	56,515	0.68 (0.21–2.15)	0.67 (0.16–1.81)
dasabuvir	0	7	3,689	56,515	NA	NA
deferasirox	9	58	3,689	56,515	2.38 (1.18–4.80)	2.26 (1.03–4.38)
diltiazem	236	4,367	3,689	56,515	0.83 (0.72–0.95)	0.85 (0.74–0.98)
dronedarone	28	501	3,689	56,515	0.86 (0.58–1.25)	0.79 (0.50–1.19)
duloxetine	122	1,916	3,689	56,515	0.98 (0.81–1.17)	0.79 (0.65–0.95)
elvitegravir	4	26	3,689	56,515	2.36 (0.82–6.76)	1.34 (0.37–3.76)
erythromycin	14	121	3,689	56,515	1.77 (1.02–3.09)	1.08 (0.57–1.90)
felbamate	0	2	3,689	56,515	NA	NA
fluconazole	57	722	3,689	56,515	1.21 (0.92–1.59)	0.96 (0.72–1.26)
fluoxetine	50	919	3,689	56,515	0.83 (0.63–1.11)	0.66 (0.49–0.87)
fluvoxamine	1	27	3,689	56,515	NA	NA
gemfibrozil	5	103	3,689	56,515	0.74 (0.30–1.83)	0.54 (0.19–1.25)
idelalisib	7	55	3,689	56,515	1.95 (0.89–4.28)	2.31 (0.95–4.77)
imatinib	4	126	3,689	56,515	0.49 (0.18–1.32)	0.54 (0.17–1.29)
indinavir	0	3	3,689	56,515	NA	NA
isavuconazole	0	8	3,689	56,515	NA	NA
itraconazole	0	30	3,689	56,515	NA	NA
ketoconazole	10	213	3,689	56,515	0.72 (0.38–1.36)	0.47 (0.23–0.87)
lopinavir	2	55	3,689	56,515	NA	NA
lorcaserin	1	7	3,689	56,515	NA	NA
methoxsalen	0	1	3,689	56,515	NA	NA
mexiletine	5	54	3,689	56,515	1.42 (0.57–3.55)	1.52 (0.53–3.48)
miconazole	3	99	3,689	56,515	0.46 (0.15–1.46)	0.36 (0.09–0.96)
mirabegron	22	557	3,689	56,515	0.61 (0.39–0.93)	0.54 (0.34–0.82)
nefazodone	0	3	3,689	56,515	NA	NA
nelfinavir	2	6	3,689	56,515	NA	NA
ombitasvir	1	12	3,689	56,515	NA	NA
paritaprevir	1	12	3,689	56,515	NA	NA
paroxetine	44	782	3,689	56,515	0.86 (0.64–1.17)	0.76 (0.55–1.02)
piperine	0	0	3,689	56,515	NA	NA
posaconazole	8	96	3,689	56,515	1.28 (0.62–2.63)	1.38 (0.61–2.68)
quinidine	0	29	3,689	56,515	NA	NA
ritonavir	16	396	3,689	56,515	0.62 (0.38–1.02)	0.56 (0.32–0.94)
rolapitant	0	4	3,689	56,515	NA	NA
saquinavir	1	2	3,689	56,515	NA	NA
telithromycin	0	1	3,689	56,515	NA	NA
terbinafine	4	124	3,689	56,515	0.49 (0.18–1.34)	0.38 (0.12–0.92)
teriflunomide	2	65	3,689	56,515	NA	NA
ticlopidine	0	9	3,689	56,515	NA	NA
tipranavir	0	2	3,689	56,515	NA	NA
vemurafenib	1	25	3,689	56,515	NA	NA
verapamil	54	990	3,689	56,515	0.84 (0.63–1.10)	0.65 (0.47–0.90)
voriconazole	1	106	3,689	56,515	NA	NA
**P-gp inhibitors**	**872**	**13,281**	**4,222**	**65,619**	**1.02 (0.95–1.10)**	**0.73 (0.66–0.81)**
amiodarone	249	6,365	4,222	65,619	0.61 (0.53–0.69)	0.55 (0.48–0.64)
clarithromycin	18	310	4,222	65,619	0.90 (0.56–1.45)	0.66 (0.39–1.04)
cobicistat	4	44	4,222	65,619	1.41 (0.51–3.93)	1.04 (0.31–2.62)
cyclosporine	30	441	4,222	65,619	1.06 (0.73–1.53)	0.83 (0.56–1.19)
digoxin	420	3,420	4,222	65,619	1.91 (1.72–2.12)	2.77 (1.77–4.61)
diltiazem	236	4,361	4,222	65,619	0.84 (0.74–0.96)	0.72 (0.50–1.00)
dronedarone	28	501	4,222	65,619	0.87 (0.59–1.27)	0.80 (0.53–1.16)
erythromycin	14	121	4,222	65,619	1.80 (1.03–3.13)	1.26 (0.69–2.15)
itraconazole	0	30	4,222	65,619	NA	NA
ketoconazole	10	213	4,222	65,619	0.73 (0.39–1.38)	0.54 (0.27–0.98)
lapatinib	1	12	4,222	65,619	NA	NA
lopinavir	2	55	4,222	65,619	NA	NA
propafenone	30	397	4,222	65,619	1.17 (0.81–1.70)	1.09 (0.73–1.56)
quinidine	0	29	4,222	65,619	NA	NA
ranolazine	14	389	4,222	65,619	0.56 (0.33–0.95)	0.52 (0.29–0.85)
ritonavir	16	396	4,222	65,619	0.63 (0.38–1.04)	0.58 (0.34–0.94)
saquinavir	1	2	4,222	65,619	NA	NA
sofosbuvir	3	80	4,222	65,619	0.58 (0.18–1.85)	0.52 (0.13–1.40)
velpatasvir	2	42	4,222	65,619	NA	NA
verapamil	54	990	4,222	65,619	0.85 (0.64–1.12)	0.75 (0.55–0.98)
voxilaprevir	0	2	4,222	65,619	NA	NA
**BBs**	**2,423**	**34,029**	**2,680**	**45,038**	**1.20 (1.13–1.27)**	**1.06 (1.00–1.13)**
acebutolol	14	85	2,680	45,038	2.77 (1.57–4.88)	2.69 (1.45–4.63)
alprenolol	1	2	2,680	45,038	NA	NA
arotinolol	0	6	2,680	45,038	NA	NA
atenolol	104	1,734	2,680	45,038	1.01 (0.82–1.23)	0.95 (0.77–1.17)
betzxolol	0	0	2,680	45,038	NA	NA
bevantolol	0	0	2,680	45,038	NA	NA
bisoprolol	864	9,717	2,680	45,038	1.49 (1.38–1.62)	1.28 (1.16–1.40)
carteolol	0	10	2,680	45,038	NA	NA
carvedilol	248	4,672	2,680	45,038	0.89 (0.78–1.02)	0.93 (0.80–1.07)
celiprolol	2	20	2,680	45,038	NA	NA
esmolol	1	8	2,680	45,038	NA	NA
labetalol	5	210	2,680	45,038	0.40 (0.16–0.97)	0.34 (0.12–0.75)
landiolol	0	9	2,680	45,038	NA	NA
levobunolol	0	7	2,680	45,038	NA	NA
metoprolol	1,103	15,066	2,680	45,038	1.23 (1.14–1.32)	1.15 (1.07–1.25)
moprolol	0	1	2,680	45,038	NA	NA
nadolol	20	126	2,680	45,038	2.67 (1.66–4.28)	2.61 (1.57–4.12)
nebivolol	64	1,014	2,680	45,038	1.06 (0.82–1.37)	1.13 (0.86–1.46)
nipradilol	0	0	2,680	45,038	NA	NA
penbutolol	0	0	2,680	45,038	NA	NA
pindolol	0	2	2,680	45,038	NA	NA
propranolol	31	516	2,680	45,038	1.01 (0.70–1.45)	0.87 (0.59–1.24)
sotalol	67	1,413	2,680	45,038	0.80 (0.62–1.02)	0.80 (0.62–1.03)
stanozolol	0	0	2,680	45,038	NA	NA
talinolol	0	0	2,680	45,038	NA	NA
timolol	29	685	2,680	45,038	0.71 (0.49–1.03)	0.72 (0.48–1.03)
**ACE inhibitors**	**1,039**	**12,562**	**4,052**	**66,313**	**1.35 (1.26–1.45)**	**1.34 (1.24–1.45)**
alacepril	0	0	4,052	66,313	NA	NA
benazepril	23	379	4,052	66,313	0.99 (0.65–1.51)	0.85 (0.54–1.27)
captopril	6	79	4,052	66,313	1.24 (0.54–2.85)	0.80 (0.31–1.72)
cilazapril	8	20	4,052	66,313	6.55 (2.88–14.87)	4.84 (1.93–11.10)
delapril	0	0	4,052	66,313	NA	NA
enalapril	59	1,092	4,052	66,313	0.88 (0.68–1.15)	0.87 (0.66–1.13)
fosinopril	0	49	4,052	66,313	NA	NA

imidapril	0	40	4,052	66,313	NA	NA
lisinopril	311	5,297	4,052	66,313	0.96 (0.85–1.08)	0.95 (0.84–1.07)
moexipril	0	0	4,052	66,313	NA	NA
pentopril	0	2	4,052	66,313	NA	NA
perindopril	376	1,552	4,052	66,313	3.96 (3.53–4.46)	3.69 (3.24–4.18)
quinapril	6	126	4,052	66,313	0.78 (0.34–1.77)	0.75 (0.29–1.57)
ramipril	259	3,978	4,052	66,313	1.07 (0.94–1.21)	1.06 (0.93–1.22)
temocapril	0	3	4,052	66,313	NA	NA
trandolapril	4	37	4,052	66,313	1.77 (0.63–4.97)	2.00 (0.59–5.09)
zofenopril	0	8	4,052	66,313	NA	NA
**ARBs**	**1,108**	**14,045**	**3,989**	**64,897**	**1.28 (1.20–1.38)**	**1.23 (1.14–1.33)**
azilsartan	9	142	3,989	64,897	1.03 (0.53–2.02)	1.13 (0.53–2.12)
candesartan	336	2,053	3,989	64,897	2.66 (2.36–3.00)	2.04 (1.78–2.34)
eprosartan	1	7	3,989	64,897	NA	NA
fimasartan	0	0	3,989	64,897	NA	NA
irbesartan	105	1,283	3,989	64,897	1.33 (1.09–1.63)	1.44 (1.17–1.76)
losartan	463	5,090	3,989	64,897	1.48 (1.34–1.64)	1.35 (1.21–1.50)
olmesartan	135	903	3,989	64,897	2.43 (2.02–2.92)	2.75 (2.27–3.31)
tasosartan	0	0	3,989	64,897	NA	NA
telmisartan	47	745	3,989	64,897	1.03 (0.76–1.38)	1.07 (0.78–1.43)
valsartan	224	4,069	3,989	64,897	0.90 (0.78–1.03)	1.02 (0.88–1.18)
**DHP-CCBs**	**1,045**	**11,704**	**4,053**	**67,211**	**1.48 (1.38–1.59)**	**1.30 (1.21–1.41)**
amlodipine	684	9,628	4,053	67,211	1.18 (1.08–1.28)	1.09 (1.00–1.19)
azelnidipine	6	50	4,053	67,211	1.99 (0.85–4.64)	2.04 (0.77–4.44)
barnidipine	2	11	4,053	67,211	NA	NA
benidipine	5	58	4,053	67,211	1.43 (0.57–3.57)	1.35 (0.47–3.08)
cilnidipine	1	67	4,053	67,211	NA	NA
clevidipine	0	0	4,053	67,211	NA	NA
felodipine	240	204	4,053	67,211	19.51 (16.14–23.58)	11.10 (8.91–13.81)
isradipine	0	11	4,053	67,211	NA	NA
lacidipine	2	16	4,053	67,211	NA	NA
lercanidipine	51	766	4,053	67,211	1.10 (0.83–1.47)	1.09 (0.81–1.44)
levamlopdipine	0	0	4,053	67,211	NA	NA
manidipine	3	16	4,053	67,211	3.11 (0.91–10.68)	2.31 (0.53–7.14)
nicardipine	2	147	4,053	67,211	NA	NA
nifedipine	56	770	4,053	67,211	1.21 (0.92–1.58)	1.09 (0.82–1.42)
nilvadipine	0	4	4,053	67,211	NA	NA
nimodipine	0	9	4,053	67,211	NA	NA
nisoldipine	0	10	4,053	67,211	NA	NA
nitrendipine	8	76	4,053	67,211	1.75 (0.84–3.62)	1.75 (0.77–3.44)
**NDHP-CCBs**	**287**	**5,321**	**4,802**	**73,507**	**0.83 (0.73–0.93)**	**0.78 (0.68–0.90)**
diltiazem	236	4,367	4,802	73,507	0.83 (0.72–0.95)	0.79 (0.68–0.92)
verapamil	54	990	4,802	73,507	0.83 (0.63–1.10)	0.72 (0.53–0.97)
**Diuretics**	**1,964**	**27,082**	**3,139**	**51,866**	**1.20 (1.13–1.27)**	**1.01 (0.95–1.08)**
althiazide	1	52	3,139	51,866	NA	NA
amiloride	9	147	3,139	51,866	1.01 (0.52–1.98)	0.94 (0.44–1.75)
azosemide	12	296	3,139	51,866	0.67 (0.38–1.19)	0.65 (0.34–1.12)
bendroflumethiazide	241	141	3,139	51,866	28.24 (22.87–34.87)	11.63 (8.97–15.08)
benzthiazide	0	3	3,139	51,866	NA	NA
buthiazide	0	1	3,139	51,866	NA	NA
chlorothiazide	3	6	3,139	51,866	8.26 (2.07–33.05)	7.76 (1.62–29.86)
chlorthalidone	20	357	3,139	51,866	0.93 (0.59–1.45)	0.83 (0.51–1.27)
clopamide	0	6	3,139	51,866	NA	NA
cyclothiazide	0	0	3,139	51,866	NA	NA
eplerenone	32	948	3,139	51,866	0.56 (0.39–0.79)	0.59 (0.40–0.82)
ethacrynic acid	1	15	3,139	51,866	NA	NA
flumethiazide	0	1	3,139	51,866	NA	NA
furosemide	841	16,284	3,139	51,866	0.85 (0.79–0.92)	0.81 (0.75–0.89)
hydrochlorothiazide	530	4,690	3,139	51,866	1.87 (1.69–2.06)	1.34 (1.19–1.50)
hydroflumethiazide	0	0	3,139	51,866	NA	NA
indapamide	88	593	3,139	51,866	2.45 (1.95–3.08)	2.01 (1.58–2.53)
methylclothiazide	0	0	3,139	51,866	NA	NA
metolazone	30	646	3,139	51,866	0.77 (0.53–1.11)	0.72 (0.49–1.03)
spironolacton	515	5,501	3,139	51,866	1.55 (1.40–1.70)	1.37 (1.23–1.53)
torsemide	262	3,985	3,139	51,866	1.09 (0.95–1.24)	1.06 (0.92–1.22)
triamterene	30	345	3,139	51,866	1.44 (0.99–2.09)	1.26 (0.85–1.81)
trichlormethiazide	7	69	3,139	51,866	1.68 (0.77–3.65)	1.50 (0.62–3.06)
xipamide	6	334	3,139	51,866	0.30 (0.13–0.67)	0.27 (0.11–0.56)
**Statins**	**1,699**	**26,488**	**3,372**	**52,040**	**0.99 (0.93–1.05)**	**0.85 (0.79–0.91)**
atorvastatin	705	14,516	3,372	52,040	0.75 (0.69–0.81)	0.64 (0.59–0.70)
cerivastatin	0	0	3,372	52,040	NA	NA
fluvastatin	5	90	3,372	52,040	0.86 (0.35–2.11)	0.66 (0.23–1.47)
lovastatin	16	442	3,372	52,040	0.56 (0.34–0.92)	0.49 (0.28–0.78)
pentostatin	0	1	3,372	52,040	NA	NA
pitavastatin	13	201	3,372	52,040	1.00 (0.57–1.75)	0.87 (0.47–1.48)
pravastatin	125	2,304	3,372	52,040	0.84 (0.70–1.01)	0.72 (0.59–0.86)
rosuvastatin	361	4,584	3,372	52,040	1.22 (1.09–1.36)	1.08 (0.96–1.22)
simvastatin	530	4,868	3,372	52,040	1.68 (1.53–1.85)	1.20 (1.08–1.34)
**Other lipid-lowering agents**	**229**	**3,319**	**4,766**	**74,592**	**1.08 (0.94–1.24)**	**0.95 (0.82–1.09)**
alirocumab	3	125	4,766	74,592	0.38 (0.12–1.18)	0.32 (0.08–0.85)
bezafibrate	5	57	4,766	74,592	1.37 (0.55–3.43)	1.14 (0.39–2.60)
ciprofibrate	0	13	4,766	74,592	NA	NA
clofibrate	0	3	4,766	74,592	NA	NA
evolocumab	14	381	4,766	74,592	0.58 (0.34–0.98)	0.49 (0.27–0.80)
ezetimibe	132	1,703	4,766	74,592	1.21 (1.01–1.45)	1.10 (0.91–1.32)
fenofibrate	41	716	4,766	74,592	0.90 (0.65–1.23)	0.76 (0.54–1.03)
gemfibrozil	5	103	4,766	74,592	0.76 (0.31–1.87)	0.54 (0.19–1.22)
linolenic acid	0	0	4,766	74,592	NA	NA
omega-3	40	418	4,766	74,592	1.50 (1.08–2.07)	1.22 (0.86–1.68)
pemafibrate	0	6	4,766	74,592	NA	NA
**Amiodarone analogs**	**276**	**6,848**	**4,761**	**71,141**	**0.60 (0.53–0.68)**	**0.59 (0.50–0.70)**
amiodarone	249	6,365	4,761	71,141	0.58 (0.51–0.67)	0.57 (0.48–0.68)
dronedarone	28	501	4,761	71,141	0.84 (0.57–1.22)	0.87 (0.57–1.29)
**Digitalis glycosides**	**441**	**3,879**	**4,543**	**73,659**	**1.84 (1.66–2.04)**	**1.72 (1.49–2.00)**
digoxin	420	3,420	4,543	73,659	1.99 (1.79–2.21)	2.14 (1.81–2.54)
digitoxin	18	460	4,543	73,659	0.63 (0.40–1.02)	0.56 (0.33–0.87)
digitalis	4	5	4,543	73,659	12.97 (3.48–48.32)	16.05 (3.94–61.20)
**Anti-platelets**	**948**	**15,139**	**4,145**	**63,806**	**0.96 (0.90–1.04)**	**0.97 (0.89–1.05)**
aspirin	779	11,725	4,145	63,806	1.02 (0.95–1.11)	1.02 (0.93–1.10)
clopidogrel	275	4,827	4,145	63,806	0.88 (0.77–0.99)	0.74 (0.63–0.86)
cilostazol	6	179	4,145	63,806	0.52 (0.23–1.16)	0.49 (0.19–1.03)
dipyridamole	1	34	4,145	63,806	NA	NA
triflusal	0	1	4,145	63,806	NA	NA
ticlopidine	0	9	4,145	63,806	NA	NA
tirofiban	0	3	4,145	63,806	NA	NA
eptifibatide	0	7	4,145	63,806	NA	NA
abciximab	0	1	4,145	63,806	NA	NA
sulodexide	0	5	4,145	63,806	NA	NA
indobufen	0	0	4,145	63,806	NA	NA
anagrelide	0	38	4,145	63,806	NA	NA
cangrelor	0	3	4,145	63,806	NA	NA
ozagrel	0	1	4,145	63,806	NA	NA
prasugrel	9	111	4,145	63,806	1.25 (0.63–2.46)	1.20 (0.55–2.27)
sarpogrelate	0	11	4,145	63,806	NA	NA
ticagrelor	16	287	4,145	63,806	0.86 (0.52–1.42)	0.86 (0.49–1.38)

Supplementary Table S3. Potential drug interactions between dabigatran and drugs of interest.

Drugs of interest	N_{11}	N_{10}	N_{01}	N_{00}	Crude ROR (95% CI)	Adjusted ROR (95% CI)
**CYP inhibitors**	360	6,111	902	13,174	0.86 (0.76–0.98)	0.92 (0.78–1.07)
abiraterone	2	41	902	13,174	NA	NA
amiodarone	68	2,014	902	13,174	0.49 (0.38–0.63)	0.50 (0.33–0.74)
aprepitant	0	16	902	13,174	NA	NA
bupropion	14	161	902	13,174	1.27 (0.73–2.20)	1.25 (0.68–2.12)
cenobamate	0	0	902	13,174	NA	NA
ceritinib	0	1	902	13,174	NA	NA
cinacalcet	0	13	902	13,174	NA	NA
ciprofloxacin	8	206	902	13,174	0.57 (0.28–1.15)	0.56 (0.25–1.07)
clarithromycin	3	83	902	13,174	0.53 (0.17–1.67)	0.53 (0.13–1.51)
clopidogrel	90	1,333	902	13,174	0.99 (0.79–1.23)	1.00 (0.76–1.32)
cobicistat	0	1	902	13,174	NA	NA
conivaptan	0	0	902	13,174	NA	NA
crizotinib	0	9	902	13,174	NA	NA
dasabuvir	0	9	902	13,174	NA	NA
deferasirox	4	18	902	13,174	3.25 (1.10–9.61)	3.21 (0.91–8.78)
diltiazem	84	1,351	902	13,174	0.91 (0.72–1.14)	0.90 (0.70–1.14)
dronedarone	28	260	902	13,174	1.57 (1.06–2.34)	1.49 (0.89–2.47)
duloxetine	21	350	902	13,174	0.88 (0.56–1.37)	0.84 (0.51–1.29)
elvitegravir	0	0	902	13,174	NA	NA
erythromycin	1	21	902	13,174	NA	NA
felbamate	0	0	902	13,174	NA	NA
fluconazole	2	84	902	13,174	NA	NA
fluoxetine	15	188	902	13,174	1.17 (0.69–1.98)	1.08 (0.60–1.79)
fluvoxamine	2	6	902	13,174	NA	NA
gemfibrozil	7	72	902	13,174	1.42 (0.65–3.09)	1.68 (0.65–3.81)
idelalisib	0	14	902	13,174	NA	NA
imatinib	1	21	902	13,174	NA	NA
indinavir	0	0	902	13,174	NA	NA
isavuconazole	0	2	902	13,174	NA	NA
itraconazole	7	9	902	13,174	11.36 (4.22–30.57)	8.70 (2.82–26.02)
ketoconazole	0	40	902	13,174	NA	NA
lopinavir	0	9	902	13,174	NA	NA
lorcaserin	0	9	902	13,174	NA	NA
methoxsalen	0	0	902	13,174	NA	NA
mexiletine	0	25	902	13,174	NA	NA
miconazole	0	26	902	13,174	NA	NA
mirabegron	11	87	902	13,174	1.85 (0.98–3.47)	1.81 (0.90–3.27)
nefazodone	0	1	902	13,174	NA	NA
nelfinavir	0	0	902	13,174	NA	NA
ombitasvir	0	10	902	13,174	NA	NA
paritaprevir	0	10	902	13,174	NA	NA
paroxetine	12	199	902	13,174	0.88 (0.49–1.58)	0.83 (0.43–1.43)
piperine	0	0	902	13,174	NA	NA
posaconazole	0	6	902	13,174	NA	NA
quinidine	2	8	902	13,174	NA	NA
ritonavir	0	48	902	13,174	NA	NA
rolapitant	0	0	902	13,174	NA	NA
saquinavir	0	0	902	13,174	NA	NA
telithromycin	0	0	902	13,174	NA	NA
terbinafine	0	22	902	13,174	NA	NA
teriflunomide	0	6	902	13,174	NA	NA
ticlopidine	0	20	902	13,174	NA	NA
tipranavir	0	0	902	13,174	NA	NA
vemurafenib	3	6	902	13,174	7.30 (1.82–29.25)	7.79 (1.61–30.41)
verapamil	24	412	902	13,174	0.85 (0.56–1.29)	0.81 (0.48–1.32)
voriconazole	0	13	902	13,174	NA	NA
**P-gp inhibitors**	285	4,974	978	14,320	0.84 (0.73–0.96)	0.78 (0.64–0.95)
amiodarone	68	2,014	978	14,320	0.49 (0.38–0.63)	0.47 (0.35–0.62)
clarithromycin	3	83	978	14,320	0.53 (0.17–1.68)	0.51 (0.12–1.39)
cobicistat	0	1	978	14,320	NA	NA
cyclosporine	5	76	978	14,320	0.96 (0.39–2.39)	0.90 (0.31–2.03)
digoxin	129	1,895	978	14,320	1.00 (0.82–1.21)	1.22 (0.53–3.54)
diltiazem	84	1,350	978	14,320	0.91 (0.72–1.15)	0.68 (0.39–1.13)
dronedarone	28	260	978	14,320	1.58 (1.06–2.34)	1.54 (0.99–2.32)
erythromycin	1	21	978	14,320	NA	NA
itraconazole	7	9	978	14,320	11.39 (4.23–30.64)	9.07 (3.09–25.60)
ketoconazole	0	40	978	14,320	NA	NA
lapatinib	0	1	978	14,320	NA	NA
lopinavir	0	9	978	14,320	NA	NA
propafenone	26	309	978	14,320	1.23 (0.82–1.85)	1.17 (0.76–1.73)
quinidine	2	8	978	14,320	NA	NA
ranolazine	12	116	978	14,320	1.51 (0.83–2.75)	1.44 (0.74–2.55)
ritonavir	0	48	978	14,320	NA	NA
saquinavir	0	0	978	14,320	NA	NA
sofosbuvir	0	18	978	14,320	NA	NA
velpatasvir	0	3	978	14,320	NA	NA
verapamil	24	412	978	14,320	0.85 (0.56–1.29)	0.87 (0.54–1.34)
voxilaprevir	0	0	978	14,320	NA	NA
**BBs**	681	9,982	591	9,348	1.08 (0.96–1.21)	1.06 (0.93–1.20)
acebutolol	0	15	591	9,348	NA	NA
alprenolol	0	0	591	9,348	NA	NA
arotinolol	0	4	591	9,348	NA	NA
atenolol	59	707	591	9,348	1.32 (1.00–1.74)	1.40 (1.04–1.85)
betzxolol	0	0	591	9,348	NA	NA
bevantolol	0	0	591	9,348	NA	NA
bisoprolol	149	2,723	591	9,348	0.87 (0.72–1.04)	0.85 (0.70–1.03)
carteolol	0	2	591	9,348	NA	NA
carvedilol	105	1,759	591	9,348	0.94 (0.76–1.17)	1.02 (0.80–1.29)
celiprolol	0	7	591	9,348	NA	NA
esmolol	0	1	591	9,348	NA	NA
labetalol	2	76	591	9,348	NA	NA
landiolol	0	1	591	9,348	NA	NA
levobunolol	0	6	591	9,348	NA	NA
metoprolol	308	3,810	591	9,348	1.28 (1.11–1.47)	1.28 (1.09–1.50)
moprolol	0	0	591	9,348	NA	NA
nadolol	2	38	591	9,348	NA	NA
nebivolol	20	345	591	9,348	0.92 (0.58–1.45)	0.83 (0.50–1.29)
nipradilol	0	0	591	9,348	NA	NA
penbutolol	0	0	591	9,348	NA	NA
pindolol	1	9	591	9,348	NA	NA
propranolol	5	145	591	9,348	0.55 (0.22–1.34)	0.57 (0.20–1.28)
sotalol	34	590	591	9,348	0.91 (0.64–1.30)	0.90 (0.61–1.28)
stanozolol	0	0	591	9,348	NA	NA
talinolol	0	0	591	9,348	NA	NA
timolol	10	132	591	9,348	1.20 (0.63–2.29)	1.39 (0.67–2.56)
**ACE inhibitors**	310	4,702	944	14,490	1.01 (0.89–1.16)	1.03 (0.89–1.20)
alacepril	0	0	944	14,490	NA	NA
benazepril	11	181	944	14,490	0.93 (0.51–1.72)	1.01 (0.51–1.81)
captopril	1	56	944	14,490	NA	NA
cilazapril	2	28	944	14,490	NA	NA
delapril	0	1	944	14,490	NA	NA
enalapril	18	483	944	14,490	0.57 (0.36–0.92)	0.61 (0.37–0.96)
fosinopril	4	31	944	14,490	1.98 (0.70–5.62)	2.26 (0.67–5.77)

imidapril	0	31	944	14,490	NA	NA
lisinopril	121	1,821	944	14,490	1.02 (0.84–1.24)	1.09 (0.88–1.34)
moexipril	0	3	944	14,490	NA	NA
pentopril	0	0	944	14,490	NA	NA
perindopril	41	610	944	14,490	1.03 (0.75–1.43)	1.08 (0.76–1.49)
quinapril	6	86	944	14,490	1.07 (0.47–2.46)	1.09 (0.42–2.32)
ramipril	105	1,367	944	14,490	1.18 (0.96–1.45)	1.16 (0.93–1.44)
temocapril	0	4	944	14,490	NA	NA
trandolapril	1	35	944	14,490	NA	NA
zofenopril	1	14	944	14,490	NA	NA
**ARBs**	281	3,933	971	15,249	1.12 (0.98–1.29)	1.14 (0.98–1.32)
azilsartan	4	45	971	15,249	1.40 (0.50–3.89)	1.70 (0.51–4.28)
candesartan	28	394	971	15,249	1.12 (0.76–1.65)	1.15 (0.75–1.67)
eprosartan	0	4	971	15,249	NA	NA
fimasartan	0	1	971	15,249	NA	NA
irbesartan	34	403	971	15,249	1.32 (0.93–1.89)	1.38 (0.94–1.96)
losartan	86	1,275	971	15,249	1.06 (0.84–1.33)	1.04 (0.82–1.32)
olmesartan	24	403	971	15,249	0.94 (0.62–1.42)	0.97 (0.62–1.45)
tasosartan	0	0	971	15,249	NA	NA
telmisartan	32	322	971	15,249	1.56 (1.08–2.26)	1.65 (1.11–2.37)
valsartan	85	1,163	971	15,249	1.15 (0.91–1.44)	1.15 (0.90–1.45)
**DHP-CCBs**	201	3,155	1,059	16,101	0.97 (0.83–1.13)	0.95 (0.80–1.11)
amlodipine	173	2,548	1,059	16,101	1.03 (0.87–1.22)	1.00 (0.83–1.18)
azelnidipine	1	35	1,059	16,101	NA	NA
barnidipine	0	16	1,059	16,101	NA	NA
benidipine	2	27	1,059	16,101	NA	NA
cilnidipine	3	25	1,059	16,101	1.82 (0.55–6.05)	2.02 (0.48–5.92)
clevidipine	0	1	1,059	16,101	NA	NA
felodipine	7	48	1,059	16,101	2.22 (1.00–4.91)	2.24 (0.92–4.68)
isradipine	0	1	1,059	16,101	NA	NA
lacidipine	0	36	1,059	16,101	NA	NA
lercanidipine	5	144	1,059	16,101	0.53 (0.22–1.29)	0.52 (0.18–1.14)
levamlopdipine	0	0	1,059	16,101	NA	NA
manidipine	0	25	1,059	16,101	NA	NA
nicardipine	0	24	1,059	16,101	NA	NA
nifedipine	12	214	1,059	16,101	0.85 (0.48–1.53)	0.88 (0.46–1.52)
nilvadipine	0	5	1,059	16,101	NA	NA
nimodipine	0	5	1,059	16,101	NA	NA
nisoldipine	1	7	1,059	16,101	NA	NA
nitrendipine	1	43	1,059	16,101	NA	NA
**NDHP-CCBs**	108	1,747	1,129	17,289	0.95 (0.77–1.16)	0.90 (0.69–1.15)
diltiazem	84	1,351	1,129	17,289	0.95 (0.76–1.20)	0.90 (0.68–1.17)
verapamil	24	412	1,129	17,289	0.89 (0.59–1.35)	0.79 (0.48–1.28)
**Diuretics**	539	7,783	724	11,491	1.10 (0.98–1.23)	1.07 (0.93–1.21)
althiazide	0	18	724	11,491	NA	NA
amiloride	1	51	724	11,491	NA	NA
azosemide	2	97	724	11,491	NA	NA
bendroflumethiazide	3	22	724	11,491	2.16 (0.65–7.25)	2.45 (0.58–7.15)
benzthiazide	0	0	724	11,491	NA	NA
buthiazide	0	0	724	11,491	NA	NA
chlorothiazide	0	4	724	11,491	NA	NA
chlorthalidone	1	78	724	11,491	NA	NA
clopamide	0	0	724	11,491	NA	NA
cyclothiazide	0	0	724	11,491	NA	NA
eplerenone	17	266	724	11,491	1.01 (0.62–1.67)	1.08 (0.62–1.75)
ethacrynic acid	0	0	724	11,491	NA	NA
flumethiazide	0	0	724	11,491	NA	NA
furosemide	352	4,983	724	11,491	1.12 (0.98–1.28)	1.09 (0.93–1.26)
hydrochlorothiazide	145	1,562	724	11,491	1.47 (1.22–1.77)	1.46 (1.18–1.79)
hydroflumethiazide	0	0	724	11,491	NA	NA
indapamide	29	243	724	11,491	1.89 (1.28–2.80)	2.12 (1.37–3.15)
methylclothiazide	0	0	724	11,491	NA	NA
metolazone	14	211	724	11,491	1.05 (0.61–1.82)	1.20 (0.65–2.04)
spironolacton	135	1,575	724	11,491	1.36 (1.12–1.65)	1.37 (1.11–1.68)
torsemide	93	792	724	11,491	1.86 (1.48–2.34)	1.80 (1.40–2.29)
triamterene	12	90	724	11,491	2.12 (1.15–3.88)	2.23 (1.14–3.99)
trichlormethiazide	2	25	724	11,491	NA	NA
xipamide	5	12	724	11,491	6.61 (2.32–18.82)	5.48 (1.72–15.08)
**Statins**	487	7,514	777	11,755	0.98 (0.87–1.10)	0.97 (0.85–1.11)
atorvastatin	225	3,498	777	11,755	0.97 (0.83–1.13)	1.00 (0.85–1.19)
cerivastatin	0	0	777	11,755	NA	NA
fluvastatin	1	47	777	11,755	NA	NA
lovastatin	12	149	777	11,755	1.22 (0.67–2.20)	1.31 (0.68–2.29)
pentostatin	0	0	777	11,755	NA	NA
pitavastatin	3	93	777	11,755	0.49 (0.15–1.54)	0.51 (0.12–1.38)
pravastatin	43	763	777	11,755	0.85 (0.62–1.17)	0.95 (0.67–1.30)
rosuvastatin	76	1,191	777	11,755	0.97 (0.76–1.23)	1.04 (0.80–1.33)
simvastatin	139	1,959	777	11,755	1.07 (0.89–1.29)	1.11 (0.91–1.35)
**Other lipid-lowering agents**	59	1,007	1,150	17,490	0.89 (0.68–1.17)	0.88 (0.66–1.15)
alirocumab	0	10	1,150	17,490	NA	NA
bezafibrate	2	31	1,150	17,490	NA	NA
ciprofibrate	1	3	1,150	17,490	NA	NA
clofibrate	0	0	1,150	17,490	NA	NA
evolocumab	1	30	1,150	17,490	NA	NA
ezetimibe	20	437	1,150	17,490	0.70 (0.44–1.09)	0.70 (0.43–1.08)
fenofibrate	18	251	1,150	17,490	1.09 (0.67–1.77)	1.06 (0.63–1.68)
gemfibrozil	7	72	1,150	17,490	1.48 (0.68–3.22)	1.32 (0.54–2.72)
linolenic acid	0	0	1,150	17,490	NA	NA
omega-3	12	233	1,150	17,490	0.78 (0.44–1.40)	0.80 (0.42–1.37)
pemafibrate	0	1	1,150	17,490	NA	NA
**Amiodarone analogs**	96	2,259	1,155	16,896	0.62 (0.50–0.77)	0.60 (0.43–0.82)
amiodarone	68	2,014	1,155	16,896	0.49 (0.39–0.63)	0.48 (0.34–0.68)
dronedarone	28	260	1,155	16,896	1.58 (1.06–2.34)	1.49 (0.92–2.34)
**Digitalis glycosides**	134	1,989	1,110	17,121	1.04 (0.86–1.25)	0.99 (0.75–1.30)
digoxin	129	1,895	1,110	17,121	1.05 (0.87–1.27)	1.02 (0.76–1.37)
digitoxin	5	91	1,110	17,121	0.85 (0.34–2.09)	0.68 (0.24–1.53)
digitalis	1	6	1,110	17,121	NA	NA
**Anti-platelets**	319	4,766	938	14,478	1.03 (0.91–1.18)	1.08 (0.94–1.25)
aspirin	267	3,878	938	14,478	1.06 (0.92–1.22)	1.10 (0.95–1.27)
clopidogrel	90	1,333	938	14,478	1.04 (0.83–1.30)	1.19 (0.88–1.60)
cilostazol	6	87	938	14,478	1.06 (0.46–2.44)	1.07 (0.42–2.27)
dipyridamole	5	51	938	14,478	1.51 (0.60–3.80)	1.67 (0.57–3.86)
triflusal	0	0	938	14,478	NA	NA
ticlopidine	0	20	938	14,478	NA	NA
tirofiban	0	0	938	14,478	NA	NA
eptifibatide	0	0	938	14,478	NA	NA
abciximab	0	1	938	14,478	NA	NA
sulodexide	0	1	938	14,478	NA	NA
indobufen	0	1	938	14,478	NA	NA
anagrelide	0	2	938	14,478	NA	NA
cangrelor	0	0	938	14,478	NA	NA
ozagrel	0	0	938	14,478	NA	NA
prasugrel	8	40	938	14,478	3.09 (1.44–6.61)	3.64 (1.56–7.48)
sarpogrelate	0	3	938	14,478	NA	NA
ticagrelor	0	82	938	14,478	NA	NA

Supplementary Table S4. Potential drug interactions between edoxaban and drugs of interest.

Drugs of interest	N_{11}	N_{10}	N_{01}	N_{00}	Crude ROR (95% CI)	Adjusted ROR (95% CI)
**CYP inhibitors**	130	1,404	293	4,498	1.42 (1.15–1.76)	0.99 (0.75–1.31)
abiraterone	6	28	293	4,498	3.29 (1.35–8.01)	4.80 (1.68–11.94)
amiodarone	58	356	293	4,498	2.50 (1.85–3.38)	3.62 (1.90–7.28)
aprepitant	2	56	293	4,498	NA	NA
bupropion	2	5	293	4,498	NA	NA
cenobamate	0	0	293	4,498	NA	NA
ceritinib	0	1	293	4,498	NA	NA
cinacalcet	1	0	293	4,498	NA	NA
ciprofloxacin	7	67	293	4,498	1.60 (0.73–3.53)	1.79 (0.73–3.74)
clarithromycin	1	54	293	4,498	NA	NA
clopidogrel	62	340	293	4,498	2.80 (2.08–3.76)	2.11 (1.30–3.47)
cobicistat	0	0	293	4,498	NA	NA
conivaptan	0	0	293	4,498	NA	NA
crizotinib	0	10	293	4,498	NA	NA
dasabuvir	0	0	293	4,498	NA	NA
deferasirox	0	7	293	4,498	NA	NA
diltiazem	1	108	293	4,498	NA	NA
dronedarone	1	23	293	4,498	NA	NA
duloxetine	1	80	293	4,498	NA	NA
elvitegravir	0	0	293	4,498	NA	NA
erythromycin	0	11	293	4,498	NA	NA
felbamate	0	0	293	4,498	NA	NA
fluconazole	1	66	293	4,498	NA	NA
fluoxetine	0	20	293	4,498	NA	NA
fluvoxamine	0	8	293	4,498	NA	NA
gemfibrozil	0	0	293	4,498	NA	NA
idelalisib	0	9	293	4,498	NA	NA
imatinib	0	9	293	4,498	NA	NA
indinavir	0	0	293	4,498	NA	NA
isavuconazole	0	0	293	4,498	NA	NA
itraconazole	0	19	293	4,498	NA	NA
ketoconazole	0	4	293	4,498	NA	NA
lopinavir	0	5	293	4,498	NA	NA
lorcaserin	0	0	293	4,498	NA	NA
methoxsalen	0	2	293	4,498	NA	NA
mexiletine	0	5	293	4,498	NA	NA
miconazole	0	11	293	4,498	NA	NA
mirabegron	2	44	293	4,498	NA	NA
nefazodone	0	0	293	4,498	NA	NA
nelfinavir	0	0	293	4,498	NA	NA
ombitasvir	0	1	293	4,498	NA	NA
paritaprevir	0	1	293	4,498	NA	NA
paroxetine	3	35	293	4,498	1.32 (0.40–4.30)	1.64 (0.39–4.78)
piperine	0	0	293	4,498	NA	NA
posaconazole	1	3	293	4,498	NA	NA
quinidine	0	0	293	4,498	NA	NA
ritonavir	2	27	293	4,498	NA	NA
rolapitant	0	0	293	4,498	NA	NA
saquinavir	0	0	293	4,498	NA	NA
telithromycin	0	0	293	4,498	NA	NA
terbinafine	0	16	293	4,498	NA	NA
teriflunomide	0	3	293	4,498	NA	NA
ticlopidine	0	5	293	4,498	NA	NA
tipranavir	0	0	293	4,498	NA	NA
vemurafenib	0	3	293	4,498	NA	NA
verapamil	15	119	293	4,498	1.94 (1.12–3.35)	3.73 (1.64–8.53)
voriconazole	3	18	293	4,498	2.56 (0.75–8.74)	3.08 (0.70–9.50)
**P-gp inhibitors**	106	953	310	4,858	1.74 (1.38–2.20)	1.45 (1.07–1.98)
amiodarone	58	356	310	4,858	2.55 (1.89–3.45)	2.73 (1.74–4.31)
clarithromycin	1	54	310	4,858	NA	NA
cobicistat	0	0	310	4,858	NA	NA
cyclosporine	0	31	310	4,858	NA	NA
digoxin	16	241	310	4,858	1.04 (0.62–1.75)	0.49 (0.23–1.01)
diltiazem	1	107	310	4,858	NA	NA
dronedarone	1	23	310	4,858	NA	NA
erythromycin	0	11	310	4,858	NA	NA
itraconazole	0	19	310	4,858	NA	NA
ketoconazole	0	5	310	4,858	NA	NA
lapatinib	1	23	310	4,858	NA	NA
lopinavir	0	5	310	4,858	NA	NA
propafenone	0	37	310	4,858	NA	NA
quinidine	0	0	310	4,858	NA	NA
ranolazine	9	43	310	4,858	3.28 (1.58–6.79)	2.66 (1.16–5.52)
ritonavir	2	27	310	4,858	NA	NA
saquinavir	0	0	310	4,858	NA	NA
sofosbuvir	1	5	310	4,858	NA	NA
velpatasvir	0	1	310	4,858	NA	NA
verapamil	15	119	310	4,858	1.98 (1.14–3.42)	2.12 (1.07–4.02)
voxilaprevir	0	0	310	4,858	NA	NA
**BBs**	232	2,761	195	3,189	1.37 (1.13–1.67)	1.17 (0.93–1.47)
acebutolol	0	0	195	3,189	NA	NA
alprenolol	0	0	195	3,189	NA	NA
arotinolol	0	2	195	3,189	NA	NA
atenolol	1	97	195	3,189	NA	NA
betzxolol	0	0	195	3,189	NA	NA
bevantolol	0	0	195	3,189	NA	NA
bisoprolol	140	1,674	195	3,189	1.37 (1.09–1.71)	1.18 (0.90–1.52)
carteolol	0	8	195	3,189	NA	NA
carvedilol	14	248	195	3,189	0.92 (0.53–1.61)	0.89 (0.47–1.58)
celiprolol	0	2	195	3,189	NA	NA
esmolol	0	0	195	3,189	NA	NA
labetalol	0	0	195	3,189	NA	NA
landiolol	0	9	195	3,189	NA	NA
levobunolol	0	0	195	3,189	NA	NA
metoprolol	100	595	195	3,189	2.75 (2.13–3.55)	2.41 (1.74–3.33)
moprolol	0	0	195	3,189	NA	NA
nadolol	0	5	195	3,189	NA	NA
nebivolol	3	81	195	3,189	0.61 (0.19–1.93)	0.57 (0.14–1.59)
nipradilol	0	1	195	3,189	NA	NA
penbutolol	0	0	195	3,189	NA	NA
pindolol	0	0	195	3,189	NA	NA
propranolol	4	33	195	3,189	1.98 (0.70–5.65)	2.45 (0.70–6.58)
sotalol	1	44	195	3,189	NA	NA
stanozolol	0	0	195	3,189	NA	NA
talinolol	0	0	195	3,189	NA	NA
timolol	0	26	195	3,189	NA	NA
**ACE inhibitors**	44	891	367	4,739	0.64 (0.46–0.88)	0.77 (0.54–1.08)
alacepril	0	0	367	4,739	NA	NA
benazepril	0	0	367	4,739	NA	NA
captopril	0	1	367	4,739	NA	NA
cilazapril	0	0	367	4,739	NA	NA
delapril	0	1	367	4,739	NA	NA
enalapril	15	161	367	4,739	1.20 (0.70–2.06)	1.41 (0.77–2.38)
fosinopril	0	2	367	4,739	NA	NA

imidapril	0	28	367	4,739	NA	NA
lisinopril	8	108	367	4,739	0.96 (0.46–1.98)	1.13 (0.50–2.26)
moexipril	0	0	367	4,739	NA	NA
pentopril	0	0	367	4,739	NA	NA
perindopril	2	86	367	4,739	NA	NA
quinapril	0	2	367	4,739	NA	NA
ramipril	19	491	367	4,739	0.50 (0.31–0.80)	0.63 (0.38–1.01)
temocapril	0	0	367	4,739	NA	NA
trandolapril	0	8	367	4,739	NA	NA
zofenopril	0	10	367	4,739	NA	NA
**ARBs**	172	1,459	251	4,417	2.07 (1.69–2.54)	1.88 (1.48–2.37)
azilsartan	4	63	251	4,417	1.12 (0.40–3.09)	1.30 (0.39–3.30)
candesartan	65	319	251	4,417	3.59 (2.67–4.82)	3.42 (2.46–4.73)
eprosartan	0	0	251	4,417	NA	NA
fimasartan	0	0	251	4,417	NA	NA
irbesartan	12	97	251	4,417	2.18 (1.18–4.02)	2.36 (1.19–4.31)
losartan	38	173	251	4,417	3.87 (2.66–5.62)	2.54 (1.59–3.95)
olmesartan	10	169	251	4,417	1.04 (0.54–2.00)	1.01 (0.49–1.88)
tasosartan	0	0	251	4,417	NA	NA
telmisartan	17	174	251	4,417	1.72 (1.03–2.87)	1.76 (0.99–2.96)
valsartan	54	491	251	4,417	1.94 (1.42–2.63)	1.46 (1.00–2.11)
**DHP-CCBs**	92	1,314	332	4,602	0.97 (0.76–1.23)	0.78 (0.61–1.01)
amlodipine	75	945	332	4,602	1.10 (0.85–1.43)	0.90 (0.68–1.17)
azelnidipine	0	20	332	4,602	NA	NA
barnidipine	0	3	332	4,602	NA	NA
benidipine	1	35	332	4,602	NA	NA
cilnidipine	0	22	332	4,602	NA	NA
clevidipine	0	0	332	4,602	NA	NA
felodipine	0	12	332	4,602	NA	NA
isradipine	0	0	332	4,602	NA	NA
lacidipine	0	9	332	4,602	NA	NA
lercanidipine	11	152	332	4,602	1.00 (0.54–1.87)	0.79 (0.39–1.47)
levamlopdipine	0	0	332	4,602	NA	NA
manidipine	0	7	332	4,602	NA	NA
nicardipine	2	11	332	4,602	NA	NA
nifedipine	2	119	332	4,602	NA	NA
nilvadipine	0	1	332	4,602	NA	NA
nimodipine	0	1	332	4,602	NA	NA
nisoldipine	0	0	332	4,602	NA	NA
nitrendipine	3	9	332	4,602	4.62 (1.24–17.15)	3.29 (0.68–12.10)
**NDHP-CCBs**	16	224	377	5,236	0.99 (0.59–1.67)	1.05 (0.92–1.21)
diltiazem	1	108	377	5,236	NA	NA
verapamil	15	119	377	5,236	1.75 (1.01–3.03)	0.00 (0.00–0.00)
**Diuretics**	196	2,504	229	3,417	1.17 (0.96–1.42)	0.79 (0.62–1.00)
althiazide	0	0	229	3,417	NA	NA
amiloride	2	6	229	3,417	NA	NA
azosemide	9	209	229	3,417	0.64 (0.33–1.27)	0.54 (0.25–1.04)
bendroflumethiazide	0	21	229	3,417	NA	NA
benzthiazide	0	0	229	3,417	NA	NA
buthiazide	0	3	229	3,417	NA	NA
chlorothiazide	0	0	229	3,417	NA	NA
chlorthalidone	0	6	229	3,417	NA	NA
clopamide	0	0	229	3,417	NA	NA
cyclothiazide	0	0	229	3,417	NA	NA
eplerenone	17	129	229	3,417	1.97 (1.17–3.32)	1.18 (0.63–2.10)
ethacrynic acid	0	0	229	3,417	NA	NA
flumethiazide	0	0	229	3,417	NA	NA
furosemide	73	1,170	229	3,417	0.93 (0.71–1.22)	0.58 (0.42–0.81)
hydrochlorothiazide	77	295	229	3,417	3.89 (2.93–5.18)	1.98 (1.39–2.79)
hydroflumethiazide	0	0	229	3,417	NA	NA
indapamide	2	40	229	3,417	NA	NA
methylclothiazide	0	0	229	3,417	NA	NA
metolazone	0	13	229	3,417	NA	NA
spironolacton	77	651	229	3,417	1.76 (1.35–2.32)	1.06 (0.75–1.48)
torsemide	105	709	229	3,417	2.21 (1.73–2.82)	1.35 (0.99–1.83)
triamterene	0	10	229	3,417	NA	NA
trichlormethiazide	2	36	229	3,417	NA	NA
xipamide	36	58	229	3,417	9.26 (5.98–14.34)	3.64 (1.94–6.66)
**Statins**	164	1,807	259	4,102	1.44 (1.17–1.76)	1.09 (0.86–1.37)
atorvastatin	85	944	259	4,102	1.43 (1.11–1.84)	1.19 (0.90–1.58)
cerivastatin	0	0	259	4,102	NA	NA
fluvastatin	1	8	259	4,102	NA	NA
lovastatin	1	2	259	4,102	NA	NA
pentostatin	0	0	259	4,102	NA	NA
pitavastatin	1	55	259	4,102	NA	NA
pravastatin	2	95	259	4,102	NA	NA
rosuvastatin	28	350	259	4,102	1.27 (0.85–1.90)	1.13 (0.72–1.70)
simvastatin	47	389	259	4,102	1.91 (1.38–2.66)	1.38 (0.92–2.01)
**Other lipid-lowering agents**	22	223	388	5,340	1.36 (0.87–2.13)	1.06 (0.65–1.66)
alirocumab	0	5	388	5,340	NA	NA
bezafibrate	10	20	388	5,340	6.88 (3.20–14.80)	6.01 (2.56–13.27)
ciprofibrate	1	1	388	5,340	NA	NA
clofibrate	0	0	388	5,340	NA	NA
evolocumab	0	9	388	5,340	NA	NA
ezetimibe	8	149	388	5,340	0.74 (0.36–1.52)	0.60 (0.26–1.17)
fenofibrate	0	30	388	5,340	NA	NA
gemfibrozil	0	0	388	5,340	NA	NA
linolenic acid	0	0	388	5,340	NA	NA
omega-3	3	16	388	5,340	2.58 (0.75–8.89)	1.58 (0.35–5.05)
pemafibrate	0	3	388	5,340	NA	NA
**Amiodarone analogs**	59	379	351	5,233	2.32 (1.73–3.12)	1.73 (1.06–2.85)
amiodarone	58	356	351	5,233	2.43 (1.80–3.27)	1.80 (1.09–2.98)
dronedarone	1	23	351	5,233	NA	NA
**Digitalis glycosides**	34	363	362	5,071	1.31 (0.91–1.89)	1.08 (0.69–1.65)
digoxin	16	241	362	5,071	0.93 (0.55–1.56)	0.57 (0.30–1.03)
digitoxin	18	120	362	5,071	2.10 (1.27–3.49)	2.03 (1.16–3.39)
digitalis	0	4	362	5,071	NA	NA
**Anti-platelets**	99	903	320	4,925	1.69 (1.33–2.14)	1.56 (1.20–2.03)
aspirin	67	619	320	4,925	1.67 (1.26–2.20)	1.44 (1.06–1.93)
clopidogrel	62	340	320	4,925	2.81 (2.09–3.76)	3.29 (2.14–5.10)
cilostazol	0	34	320	4,925	NA	NA
dipyridamole	0	0	320	4,925	NA	NA
triflusal	0	0	320	4,925	NA	NA
ticlopidine	0	5	320	4,925	NA	NA
tirofiban	0	0	320	4,925	NA	NA
eptifibatide	0	0	320	4,925	NA	NA
abciximab	0	0	320	4,925	NA	NA
sulodexide	0	0	320	4,925	NA	NA
indobufen	0	0	320	4,925	NA	NA
anagrelide	0	16	320	4,925	NA	NA
cangrelor	0	0	320	4,925	NA	NA
ozagrel	1	3	320	4,925	NA	NA
prasugrel	0	50	320	4,925	NA	NA
sarpogrelate	1	7	320	4,925	NA	NA
ticagrelor	0	15	320	4,925	NA	NA

Supplementary Table S5. Potential drug interactions between rivaroxaban and drugs of interest.

Drugs of interest	N_{11}	N_{10}	N_{01}	N_{00}	Crude ROR (95% CI)	Adjusted ROR (95% CI)
**CYP inhibitors**	1,292	20,017	3,865	56,460	0.94 (0.88–1.01)	0.92 (0.86–1.00)
abiraterone	16	209	3,865	56,460	1.12 (0.67–1.86)	1.31 (0.75–2.12)
amiodarone	221	4,578	3,865	56,460	0.71 (0.61–0.81)	0.72 (0.57–0.91)
aprepitant	11	141	3,865	56,460	1.14 (0.62–2.11)	1.02 (0.52–1.82)
bupropion	44	770	3,865	56,460	0.83 (0.61–1.13)	0.72 (0.52–0.97)
cenobamate	0	0	3,865	56,460	NA	NA
ceritinib	2	17	3,865	56,460	NA	NA
cinacalcet	2	60	3,865	56,460	NA	NA
ciprofloxacin	88	742	3,865	56,460	1.73 (1.39–2.17)	1.39 (1.10–1.75)
clarithromycin	56	271	3,865	56,460	3.02 (2.26–4.03)	2.44 (1.71–3.45)
clopidogrel	435	6,420	3,865	56,460	0.99 (0.89–1.10)	1.02 (0.91–1.14)
cobicistat	0	34	3,865	56,460	NA	NA
conivaptan	0	0	3,865	56,460	NA	NA
crizotinib	10	88	3,865	56,460	1.66 (0.86–3.20)	1.64 (0.79–3.00)
dasabuvir	0	12	3,865	56,460	NA	NA
deferasirox	8	41	3,865	56,460	2.85 (1.34–6.08)	2.86 (1.24–5.82)
diltiazem	247	3,246	3,865	56,460	1.11 (0.97–1.27)	1.05 (0.91–1.20)
dronedarone	33	530	3,865	56,460	0.91 (0.64–1.29)	0.88 (0.58–1.31)
duloxetine	95	1,431	3,865	56,460	0.97 (0.79–1.20)	0.81 (0.65–1.01)
elvitegravir	0	20	3,865	56,460	NA	NA
erythromycin	12	98	3,865	56,460	1.79 (0.98–3.26)	1.25 (0.63–2.29)
felbamate	0	0	3,865	56,460	NA	NA
fluconazole	81	499	3,865	56,460	2.37 (1.87–3.01)	1.77 (1.37–2.25)
fluoxetine	43	857	3,865	56,460	0.73 (0.54–1.00)	0.64 (0.46–0.87)
fluvoxamine	2	23	3,865	56,460	NA	NA
gemfibrozil	13	146	3,865	56,460	1.30 (0.74–2.30)	1.39 (0.73–2.47)
idelalisib	3	53	3,865	56,460	0.83 (0.26–2.65)	0.80 (0.19–2.19)
imatinib	3	88	3,865	56,460	0.50 (0.16–1.57)	0.50 (0.12–1.34)
indinavir	0	2	3,865	56,460	NA	NA
isavuconazole	0	2	3,865	56,460	NA	NA
itraconazole	0	31	3,865	56,460	NA	NA
ketoconazole	5	122	3,865	56,460	0.60 (0.24–1.47)	0.48 (0.17–1.09)
lopinavir	0	28	3,865	56,460	NA	NA
lorcaserin	0	10	3,865	56,460	NA	NA
methoxsalen	0	3	3,865	56,460	NA	NA
mexiletine	2	36	3,865	56,460	NA	NA
miconazole	3	59	3,865	56,460	0.74 (0.23–2.37)	0.64 (0.16–1.74)
mirabegron	20	386	3,865	56,460	0.76 (0.48–1.19)	0.68 (0.42–1.05)
nefazodone	0	5	3,865	56,460	NA	NA
nelfinavir	0	2	3,865	56,460	NA	NA
ombitasvir	0	11	3,865	56,460	NA	NA
paritaprevir	0	11	3,865	56,460	NA	NA
paroxetine	32	747	3,865	56,460	0.63 (0.44–0.89)	0.58 (0.39–0.81)
piperine	0	0	3,865	56,460	NA	NA
posaconazole	4	36	3,865	56,460	1.62 (0.58–4.56)	1.71 (0.51–4.29)
quinidine	2	30	3,865	56,460	NA	NA
ritonavir	7	124	3,865	56,460	0.82 (0.38–1.77)	0.82 (0.34–1.70)
rolapitant	0	1	3,865	56,460	NA	NA
saquinavir	0	0	3,865	56,460	NA	NA
telithromycin	0	0	3,865	56,460	NA	NA
terbinafine	2	88	3,865	56,460	NA	NA
teriflunomide	3	60	3,865	56,460	0.73 (0.23–2.33)	0.68 (0.16–1.91)
ticlopidine	0	18	3,865	56,460	NA	NA
tipranavir	0	1	3,865	56,460	NA	NA
vemurafenib	3	28	3,865	56,460	1.57 (0.48–5.15)	1.61 (0.38–4.56)
verapamil	51	876	3,865	56,460	0.85 (0.64–1.13)	0.76 (0.54–1.06)
voriconazole	8	77	3,865	56,460	1.52 (0.73–3.15)	1.46 (0.64–2.86)
**P-gp inhibitors**	674	10,764	4,481	65,658	0.92 (0.84–1.00)	0.93 (0.83–1.04)
amiodarone	221	4,578	4,481	65,658	0.71 (0.62–0.81)	0.75 (0.64–0.87)
clarithromycin	56	271	4,481	65,658	3.03 (2.27–4.04)	2.74 (2.01–3.67)
cobicistat	0	34	4,481	65,658	NA	NA
cyclosporine	24	264	4,481	65,658	1.33 (0.88–2.03)	1.16 (0.74–1.74)
digoxin	218	3,194	4,481	65,658	1.00 (0.87–1.15)	2.08 (1.15–4.24)
diltiazem	247	3,243	4,481	65,658	1.12 (0.98–1.27)	1.08 (0.77–1.48)
dronedarone	33	530	4,481	65,658	0.91 (0.64–1.30)	0.96 (0.66–1.36)
erythromycin	12	98	4,481	65,658	1.79 (0.98–3.27)	1.48 (0.76–2.61)
itraconazole	0	31	4,481	65,658	NA	NA
ketoconazole	5	122	4,481	65,658	0.60 (0.25–1.47)	0.52 (0.18–1.14)
lapatinib	2	32	4,481	65,658	NA	NA
lopinavir	0	28	4,481	65,658	NA	NA
propafenone	32	450	4,481	65,658	1.04 (0.73–1.49)	1.04 (0.71–1.46)
quinidine	2	30	4,481	65,658	NA	NA
ranolazine	27	413	4,481	65,658	0.96 (0.65–1.42)	1.00 (0.66–1.46)
ritonavir	7	124	4,481	65,658	0.83 (0.39–1.77)	0.88 (0.37–1.77)
saquinavir	0	0	4,481	65,658	NA	NA
sofosbuvir	6	73	4,481	65,658	1.20 (0.52–2.77)	1.24 (0.48–2.64)
velpatasvir	3	16	4,481	65,658	2.75 (0.80–9.43)	2.99 (0.69–9.07)
verapamil	51	876	4,481	65,658	0.85 (0.64–1.13)	0.88 (0.65–1.17)
voxilaprevir	0	3	4,481	65,658	NA	NA
**BBs**	1,679	25,775	3,479	50,675	0.95 (0.89–1.01)	0.93 (0.87–1.00)
acebutolol	2	137	3,479	50,675	NA	NA
alprenolol	0	1	3,479	50,675	NA	NA
arotinolol	0	5	3,479	50,675	NA	NA
atenolol	155	1,890	3,479	50,675	1.19 (1.01–1.41)	1.14 (0.96–1.35)
betzxolol	0	0	3,479	50,675	NA	NA
bevantolol	0	0	3,479	50,675	NA	NA
bisoprolol	373	6,831	3,479	50,675	0.80 (0.71–0.89)	0.83 (0.73–0.93)
carteolol	0	20	3,479	50,675	NA	NA
carvedilol	245	3,801	3,479	50,675	0.94 (0.82–1.07)	0.97 (0.83–1.12)
celiprolol	1	14	3,479	50,675	NA	NA
esmolol	0	9	3,479	50,675	NA	NA
labetalol	11	186	3,479	50,675	0.86 (0.47–1.58)	0.77 (0.39–1.35)
landiolol	0	1	3,479	50,675	NA	NA
levobunolol	0	5	3,479	50,675	NA	NA
metoprolol	727	10,738	3,479	50,675	0.99 (0.91–1.07)	0.98 (0.89–1.07)
moprolol	0	0	3,479	50,675	NA	NA
nadolol	11	108	3,479	50,675	1.48 (0.80–2.76)	1.34 (0.68–2.40)
nebivolol	50	917	3,479	50,675	0.79 (0.60–1.06)	0.82 (0.60–1.08)
nipradilol	0	0	3,479	50,675	NA	NA
penbutolol	0	0	3,479	50,675	NA	NA
pindolol	0	19	3,479	50,675	NA	NA
propranolol	64	439	3,479	50,675	2.12 (1.63–2.77)	1.89 (1.43–2.46)
sotalol	82	1,221	3,479	50,675	0.98 (0.78–1.23)	0.97 (0.76–1.21)
stanozolol	0	1	3,479	50,675	NA	NA
talinolol	0	3	3,479	50,675	NA	NA
timolol	19	470	3,479	50,675	0.59 (0.37–0.93)	0.58 (0.35–0.90)
**ACE inhibitors**	785	11,957	4,369	64,449	0.97 (0.90–1.05)	0.96 (0.88–1.04)
alacepril	0	3	4,369	64,449	NA	NA
benazepril	35	437	4,369	64,449	1.18 (0.84–1.67)	1.10 (0.76–1.54)
captopril	4	55	4,369	64,449	1.07 (0.39–2.96)	1.04 (0.31–2.55)
cilazapril	1	6	4,369	64,449	NA	NA
delapril	0	2	4,369	64,449	NA	NA
enalapril	60	912	4,369	64,449	0.97 (0.75–1.26)	0.96 (0.73–1.24)
fosinopril	11	66	4,369	64,449	2.46 (1.30–4.66)	2.52 (1.25–4.60)

imidapril	1	49	4,369	64,449	NA	NA
lisinopril	348	5,571	4,369	64,449	0.92 (0.82–1.03)	0.90 (0.79–1.01)
moexipril	0	10	4,369	64,449	NA	NA
pentopril	0	0	4,369	64,449	NA	NA
perindopril	61	1,319	4,369	64,449	0.68 (0.53–0.88)	0.72 (0.54–0.92)
quinapril	18	183	4,369	64,449	1.45 (0.89–2.36)	1.46 (0.87–2.32)
ramipril	257	3,321	4,369	64,449	1.14 (1.00–1.30)	1.17 (1.02–1.34)
temocapril	0	5	4,369	64,449	NA	NA
trandolapril	1	101	4,369	64,449	NA	NA
zofenopril	2	28	4,369	64,449	NA	NA
**ARBs**	729	10,715	4,413	65,510	1.01 (0.93–1.10)	0.99 (0.90–1.08)
azilsartan	4	102	4,413	65,510	0.58 (0.21–1.58)	0.54 (0.17–1.30)
candesartan	91	1,528	4,413	65,510	0.88 (0.71–1.09)	0.88 (0.70–1.08)
eprosartan	1	14	4,413	65,510	NA	NA
fimasartan	1	0	4,413	65,510	NA	NA
irbesartan	77	933	4,413	65,510	1.23 (0.97–1.55)	1.19 (0.93–1.50)
losartan	252	3,752	4,413	65,510	1.00 (0.87–1.14)	0.94 (0.81–1.07)
olmesartan	47	833	4,413	65,510	0.84 (0.62–1.13)	0.76 (0.56–1.02)
tasosartan	0	0	4,413	65,510	NA	NA
telmisartan	65	712	4,413	65,510	1.36 (1.05–1.75)	1.28 (0.98–1.65)
valsartan	200	3,087	4,413	65,510	0.96 (0.83–1.11)	0.94 (0.81–1.10)
**DHP-CCBs**	654	9,105	4,480	66,943	1.07 (0.99–1.17)	1.06 (0.97–1.16)
amlodipine	526	7,400	4,480	66,943	1.06 (0.97–1.17)	1.04 (0.94–1.15)
azelnidipine	1	41	4,480	66,943	NA	NA
barnidipine	1	19	4,480	66,943	NA	NA
benidipine	2	43	4,480	66,943	NA	NA
cilnidipine	0	64	4,480	66,943	NA	NA
clevidipine	0	0	4,480	66,943	NA	NA
felodipine	9	161	4,480	66,943	0.84 (0.43–1.64)	0.87 (0.41–1.60)
isradipine	1	10	4,480	66,943	NA	NA
lacidipine	6	28	4,480	66,943	3.20 (1.33–7.74)	3.13 (1.16–7.12)
lercanidipine	55	628	4,480	66,943	1.31 (0.99–1.73)	1.36 (1.02–1.79)
levamlopdipine	0	0	4,480	66,943	NA	NA
manidipine	1	36	4,480	66,943	NA	NA
nicardipine	2	80	4,480	66,943	NA	NA
nifedipine	55	640	4,480	66,943	1.28 (0.97–1.69)	1.17 (0.88–1.54)
nilvadipine	0	1	4,480	66,943	NA	NA
nimodipine	0	10	4,480	66,943	NA	NA
nisoldipine	0	11	4,480	66,943	NA	NA
nitrendipine	2	51	4,480	66,943	NA	NA
**NDHP-CCBs**	296	4,095	4,840	72,060	1.08 (0.95–1.22)	0.75 (0.66–0.86)
diltiazem	247	3,246	4,840	72,060	1.13 (0.99–1.29)	1.13 (0.97–1.31)
verapamil	51	876	4,840	72,060	0.87 (0.65–1.15)	0.75 (0.54–1.01)
**Diuretics**	1,387	19,785	3,770	56,635	1.05 (0.99–1.12)	1.04 (0.96–1.12)
althiazide	1	32	3,770	56,635	NA	NA
amiloride	8	116	3,770	56,635	1.04 (0.51–2.12)	1.01 (0.45–1.96)
azosemide	8	218	3,770	56,635	0.55 (0.27–1.12)	0.52 (0.23–0.99)
bendroflumethiazide	26	136	3,770	56,635	2.87 (1.89–4.37)	2.52 (1.61–3.80)
benzthiazide	0	1	3,770	56,635	NA	NA
buthiazide	0	5	3,770	56,635	NA	NA
chlorothiazide	0	11	3,770	56,635	NA	NA
chlorthalidone	16	275	3,770	56,635	0.87 (0.53–1.45)	0.85 (0.49–1.37)
clopamide	0	0	3,770	56,635	NA	NA
cyclothiazide	0	0	3,770	56,635	NA	NA
eplerenone	53	647	3,770	56,635	1.23 (0.93–1.63)	1.37 (1.01–1.82)
ethacrynic acid	1	14	3,770	56,635	NA	NA
flumethiazide	0	0	3,770	56,635	NA	NA
furosemide	776	11,508	3,770	56,635	1.01 (0.94–1.10)	1.00 (0.92–1.10)
hydrochlorothiazide	343	4,346	3,770	56,635	1.19 (1.06–1.33)	1.15 (1.02–1.31)
hydroflumethiazide	0	0	3,770	56,635	NA	NA
indapamide	58	503	3,770	56,635	1.73 (1.32–2.28)	1.77 (1.32–2.32)
methylclothiazide	0	0	3,770	56,635	NA	NA
metolazone	26	399	3,770	56,635	0.98 (0.66–1.46)	0.91 (0.59–1.34)
spironolacton	308	3,806	3,770	56,635	1.22 (1.08–1.37)	1.22 (1.07–1.39)
torsemide	169	2,489	3,770	56,635	1.02 (0.87–1.20)	1.03 (0.87–1.21)
triamterene	42	340	3,770	56,635	1.86 (1.34–2.56)	1.65 (1.17–2.26)
trichlormethiazide	4	60	3,770	56,635	1.00 (0.36–2.76)	0.88 (0.26–2.15)
xipamide	2	88	3,770	56,635	NA	NA
**Statins**	1,336	20,278	3,808	55,932	0.97 (0.91–1.03)	0.94 (0.87–1.01)
atorvastatin	635	9,708	3,808	55,932	0.96 (0.88–1.05)	0.95 (0.86–1.05)
cerivastatin	0	0	3,808	55,932	NA	NA
fluvastatin	7	64	3,808	55,932	1.61 (0.74–3.51)	1.56 (0.65–3.19)
lovastatin	29	459	3,808	55,932	0.93 (0.64–1.35)	0.88 (0.59–1.27)
pentostatin	0	0	3,808	55,932	NA	NA
pitavastatin	13	181	3,808	55,932	1.05 (0.60–1.85)	1.02 (0.55–1.73)
pravastatin	102	2,060	3,808	55,932	0.73 (0.59–0.89)	0.71 (0.58–0.87)
rosuvastatin	264	3,414	3,808	55,932	1.14 (1.00–1.29)	1.14 (0.99–1.30)
simvastatin	310	4,912	3,808	55,932	0.93 (0.82–1.04)	0.92 (0.81–1.05)
**Other lipid-lowering agents**	209	2,972	4,861	71,946	1.04 (0.90–1.20)	1.01 (0.87–1.17)
alirocumab	1	50	4,861	71,946	NA	NA
bezafibrate	0	46	4,861	71,946	NA	NA
ciprofibrate	0	9	4,861	71,946	NA	NA
clofibrate	0	5	4,861	71,946	NA	NA
evolocumab	6	190	4,861	71,946	0.47 (0.21–1.05)	0.46 (0.18–0.94)
ezetimibe	112	1,438	4,861	71,946	1.15 (0.95–1.40)	1.15 (0.94–1.39)
fenofibrate	55	805	4,861	71,946	1.01 (0.77–1.33)	0.97 (0.72–1.26)
gemfibrozil	13	146	4,861	71,946	1.32 (0.75–2.33)	1.23 (0.66–2.10)
linolenic acid	0	0	4,861	71,946	NA	NA
omega-3	26	461	4,861	71,946	0.83 (0.56–1.24)	0.76 (0.50–1.11)
pemafibrate	0	6	4,861	71,946	NA	NA
**Amiodarone analogs**	251	5,079	4,844	70,626	0.72 (0.63–0.82)	0.71 (0.59–0.86)
amiodarone	221	4,578	4,844	70,626	0.70 (0.61–0.81)	0.70 (0.57–0.84)
dronedarone	33	530	4,844	70,626	0.91 (0.64–1.29)	0.85 (0.57–1.22)
**Digitalis glycosides**	228	3,523	4,862	72,020	0.96 (0.84–1.10)	0.87 (0.73–1.03)
digoxin	218	3,194	4,862	72,020	1.01 (0.88–1.16)	0.94 (0.78–1.13)
digitoxin	11	310	4,862	72,020	0.53 (0.29–0.96)	0.51 (0.26–0.89)
digitalis	1	25	4,862	72,020	NA	NA
**Anti-platelets**	1,965	29,614	3,185	46,797	0.97 (0.92–1.03)	1.06 (1.00–1.13)
aspirin	1,795	26,944	3,185	46,797	0.98 (0.92–1.04)	1.05 (0.99–1.12)
clopidogrel	435	6,420	3,185	46,797	1.00 (0.90–1.10)	1.23 (1.07–1.42)
cilostazol	12	240	3,185	46,797	0.73 (0.41–1.31)	0.78 (0.41–1.33)
dipyridamole	7	80	3,185	46,797	1.29 (0.59–2.79)	1.38 (0.58–2.80)
triflusal	3	1	3,185	46,797	NA	NA
ticlopidine	0	18	3,185	46,797	NA	NA
tirofiban	0	6	3,185	46,797	NA	NA
eptifibatide	0	1	3,185	46,797	NA	NA
abciximab	0	3	3,185	46,797	NA	NA
sulodexide	1	5	3,185	46,797	NA	NA
indobufen	0	1	3,185	46,797	NA	NA
anagrelide	1	35	3,185	46,797	NA	NA
cangrelor	2	3	3,185	46,797	NA	NA
ozagrel	0	4	3,185	46,797	NA	NA
prasugrel	16	256	3,185	46,797	0.92 (0.55–1.52)	1.03 (0.60–1.66)
sarpogrelate	1	13	3,185	46,797	NA	NA
ticagrelor	28	403	3,185	46,797	1.02 (0.69–1.50)	1.11 (0.74–1.61)

Supplementary Table S6. Definition of upper gastrointestinal adverse events.

Definition	LLTs
UGI discomfort	Abdominal cramp; Abdominal cramps; Abdominal crampy pains; Abdominal discomfort; Abdominal distress; Abdominal pain; Abdominal pain aggravated; Abdominal pain generalised; Abdominal pain generalized; Abdominal pain localised; Abdominal pain localized; Abdominal pain NOS; Abdominal pain upper; Abdominal symptom; Abdominal symptom NOS; Abdominal tenderness; Abdominal wall strained; Ache stomach; Acute gastric pain; Belly ache; Central abdominal pain; Chronic abdominal pain; Chronic epigastric pain; Cramp abdominal; Discomfort abdominal; Discomfort epigastric; Distress abdominal; Distress epigastric; Distress gastrointestinal; Epigastralgia; Epigastric ache; Epigastric cramp; Epigastric discomfort; Epigastric distress; Epigastric food-related pain; Epigastric pain; Epigastric pain not food-related; Esophagalgia; Esophageal discomfort; Esophageal irritation; Esophageal pain; Functional abdominal pain; Functional gastrointestinal disorder; Gastralgia; Gastric irritation; Gastric pain; Gastric spasm; Gastrointestinal cramps; Gastrointestinal discomfort; Gastrointestinal disorder therapy; Gastrointestinal irritation; Gastrointestinal pain; Gastrointestinal pain NOS; Gastrointestinal spasm; Gastrointestinal stinging; Gastrointestinal tract irritation; Gastrointestinal upset; GI irritation; GI pain; GI upset; Griping abdomen; Griping abdominal; Gut pain; Irritable stomach; Irritation gastric; Irritation gastrointestinal; Left upper abdominal discomfort; Left upper quadrant pain; Mesogastric pain; Oesophageal discomfort; Oesophageal irritation; Oesophageal pain; Pain abdominal; Pain epigastric; Pain esophageal; Pain gastric; Pain oesophageal; Pain right upper quadrant; Pain stomach; Periumbilical pain; Postprandial pain; Reactive gastropathy; Right upper abdominal discomfort; Right upper quadrant pain; RUQ pain; Sore esophagus; Sore oesophagus; Sour stomach; Stomach ache; Stomach cramps; Stomach discomfort; Stomach dull pain of; Stomach feeling heavy; Stomach heaviness; Stomach irritated feeling of; Stomach pain; Stomach upset; Stomachache; Tenderness epigastric; Tummy ache; Upper abdominal discomfort; Upper abdominal pain; Upper abdominal pressure sensation; Upper abdominal tenderness; Upset gastrointestinal; Upset stomach
Dyspepsia	Acid dyspepsia; Acid indigestion; Delayed gastric emptying; Difficult digestion; Digestion impaired; Digestive enzyme abnormal; Digestive enzyme decreased; Dyspepsia; Dyspepsia aggravated; Feeling queasy; Flatulent dyspepsia; Functional dyspepsia; Increasing indigestion; Indigestion; Indigestion acid; Indigestion nervous; Maldigestion; Non-ulcer dyspepsia; Pepsinogen I decreased; Pepsinogen I increased; Ulcer-like dyspepsia;
Reflux	Acid peptic disease; Acid reflux (esophageal); Acid reflux (oesophageal); Acute erosive duodenitis; Acute gastric erosions; Antacid therapy; Burning esophagus; Burning in abdomen; Burning oesophagus; Burning sensation in abdomen; Chest burning; Chest burning pain of; Chronic erosive gastritis; Chronic heartburn; Duodenal erosion; Duodenal erosions; Duodenogastric reflux; Duodenogastroesophageal reflux; Duodenogastrooesophageal reflux; Endoscopy negative reflux disease; Epigastric burning; Erosive duodenitis; Erosive esophagitis; Erosive gastroduodenitis; Erosive oesophagitis; Esophageal acid reflux; Esophageal erosion; Esophageal erosions; Esophageal reflux; Esophageal reflux aggravated; Gastric erosions; Gastric mucosa erosion; Gastritis alkaline reflux; Gastritis erosive; Gastroesophageal burning; Gastroesophageal reflux; Gastroesophageal reflux disease; Gastroesophageal reflux prophylaxis; Gastrointestinal erosion; Gastrooesophageal burning; Gastrooesophageal reflux; Gastrooesophageal reflux disease; Gastrooesophageal reflux prophylaxis; GERD; GORD; Gum erosion; Heartburn; Heartburn aggravated; Heartburn-like dyspepsia; Lip erosion; Mucosa erosion crust; Mucosal erosion; Mucosal erosion NOS; Multiple gastric erosions; Non-erosive reflux disease; Oesophageal acid reflux; Oesophageal erosion; Oesophageal erosions; Oesophageal reflux; Oesophageal reflux aggravated; Pharyngeal erosion; Pyrosis; Reflux esophagitis; Reflux gastritis; Reflux oesophagitis; Retrosternal burning; Stomach burning sensation of; Stomach erosion; Waterbrash
Ulcer	Acute duodenal ulcer with haemorrhage; Acute duodenal ulcer with haemorrhage, without mention of obstruction; Acute duodenal ulcer with hemorrhage; Acute duodenal ulcer with hemorrhage, without mention of obstruction; Acute duodenal ulcer without mention of haemorrhage or perforation; Acute duodenal ulcer without mention of hemorrhage or perforation; Acute gastric ulcer with haemorrhage; Acute gastric ulcer with haemorrhage, with obstruction; Acute gastric ulcer with haemorrhage, without mention of obstruction; Acute gastric ulcer with hemorrhage; Acute gastric ulcer with hemorrhage, with obstruction; Acute gastric ulcer with hemorrhage, without mention of obstruction; Acute gastric ulcer without mention of haemorrhage or perforation; Acute gastric ulcer without mention of hemorrhage or perforation; Acute gastrojejunal ulcer with haemorrhage; Acute gastrojejunal ulcer with haemorrhage, without mention of obstruction; Acute gastrojejunal ulcer with hemorrhage; Acute gastrojejunal ulcer with hemorrhage, without mention of obstruction; Acute gastrojejunal ulcer, with haemorrhage, with obstruction; Acute gastrojejunal ulcer, with hemorrhage, with obstruction; Acute peptic ulcer of unspecified site with haemorrhage; Acute peptic ulcer of unspecified site with hemorrhage; Gastrojejunal ulcer, acute with haemorrhage; Chronic or unspecified gastrojejunal ulcer with hemorrhage; Chronic gastrojejunal ulcer without mention of haemorrhage or perforation; Chronic gastrojejunal ulcer without mention of hemorrhage or perforation; Chronic or unspecified gastrojejunal ulcer with haemorrhage; Gastrojejunal ulcer, acute with hemorrhage; Acute gastrojejunal ulcer without mention of hemorrhage or perforation; Acute gastrojejunal ulcer without mention of haemorrhage or perforation; Chronic duodenal ulcer without mention of haemorrhage or perforation; Chronic duodenal ulcer without mention of hemorrhage or perforation; Chronic gastric ulcer without mention of haemorrhage or perforation; Chronic gastric ulcer without mention of hemorrhage or perforation; Chronic or unspecified gastric ulcer with haemorrhage; Chronic or unspecified gastric ulcer with hemorrhage; Chronic or unspecified peptic ulcer of unspecified site with haemorrhage; Chronic or unspecified peptic ulcer of unspecified site with hemorrhage; Duodenal ulcer haemorrhage; Duodenal ulcer hemorrhage; Duodenal ulcer, acute with haemorrhage; Duodenal ulcer, acute with hemorrhage; Esophageal ulcer hemorrhage; Esophageal ulceration hemorrhage; Esophagus ulceration hemorrhage; Gastric ulcer chronic or unspecified with haemorrhage; Gastric ulcer chronic or unspecified with hemorrhage; Gastric ulcer haemorrhage; Gastric ulcer haemorrhage, obstructive; Gastric ulcer hemorrhage; Gastric ulcer hemorrhage, obstructive; Gastric ulcer, acute with haemorrhage; Gastric ulcer, acute with hemorrhage; Gastroesophageal junction ulcer hemorrhage; Gastrointestinal ulcer haemorrhage; Gastrointestinal ulcer hemorrhage; Gastrooesophageal junction ulcer haemorrhage; Oesophageal ulcer haemorrhage; Oesophageal ulceration haemorrhage; Oesophagus ulceration haemorrhage; Peptic ulcer haemorrhage; Peptic ulcer hemorrhage; Stomach ulcer haemorrhage; Stomach ulcer hemorrhage; Stomach ulcer with haemorrhage; Stomach ulcer with hemorrhage; Stress ulcer haemorrhage; Stress ulcer hemorrhage; Ulcer duodenal haemorrhage; Ulcer duodenal hemorrhage; Ulcer haemorrhage; Ulcer haemorrhage NOS; Ulcer hemorrhage; Ulcer peptic with haemorrhage; Ulcer peptic with hemorrhage; Ulcer stomach with haemorrhage; Ulcer stomach with hemorrhage; Abdominal pain peptic ulcer type; Acute duodenal ulcer; Acute duodenal ulcer with perforation; Acute duodenal ulcer with perforation, with obstruction; Acute duodenal ulcer with perforation, without mention of obstruction; Acute erosive duodenitis; Acute gastric erosions; Acute gastric ulcer with perforation; Acute gastric ulcer with perforation, with obstruction; Acute gastric ulcer with perforation, without mention of obstruction; Acute haemorrhagic ulcerative colitis; Acute hemorrhagic ulcerative colitis; Acute peptic ulcer of unspecified site with perforation; Acute peptic ulcer of unspecified site with perforation, with obstruction; Antral ulcer; Acute gastrojejunal ulcer with perforation; Gastrojejunal ulcer; Ulcer gastrojejunal; Bleeding duodenal ulcer; Bleeding esophageal ulcer; Bleeding gastric ulcer; Bleeding oesophageal ulcer; Bleeding peptic ulcer; Gastrojejunal ulcer, acute with perforation; Duodenal ulcer bleeding; Acute gastrojejunal ulcer with perforation, without mention of obstruction; Cameron ulcer; Gastro-jejunal ulcer; Gastric ulcer bleeding; Chronic erosive gastritis; Chronic peptic ulcer; Curling's ulcer; Cushing's ulcer; Cytomegalovirus gastrointestinal ulcer; Duodenal erosion; Duodenal erosions; Duodenal scarring; Duodenal ulcer; Duodenal ulcer aggravated; Gastric ulcer bleeding, obstructive; Duodenal ulcer haemorrhagic; Duodenal ulcer hemorrhagic; Duodenal ulcer perforated; Duodenal ulcer perforation; Duodenal ulcer perforation, obstructive; Duodenal ulcer reactivated; Duodenal ulcer repair; Duodenal ulcer, obstructive; Duodenal ulcer-type symptoms; Enanthematic gastritis; Erosive duodenitis; Erosive esophagitis; Erosive gastroduodenitis; Erosive oesophagitis; Esophageal erosion; Esophageal erosions; Esophageal ulcer; Esophageal ulcer perforation; Esophageal ulceration; Esophagitis ulcerative; Esophagus ulceration; Gastric aphthous ulcer; Gastric erosions; Gastric mucosa erosion; Gastric ulcer; Gastric ulcer acute, with perforation; Gastrointestinal ulcer bleeding; Gastric ulcer haemorrhagic; Gastric ulcer helicobacter; Gastric ulcer hemorrhagic; Gastric ulcer perforated; Gastric ulcer perforation; Gastric ulcer perforation, obstructive; Gastric ulcer prophylaxis; Gastric ulcer reactivation; Gastric ulcer surgery; Gastric ulcer, obstructive; Gastritis erosive; Gastritis hypertrophic; Gastroduodenal ulcer; Gastroesophageal junction ulcer; Gastrointestinal cramps; Gastrointestinal erosion; Gastrointestinal pain; Gastrointestinal pain NOS; Gastrointestinal scarring; Gastrointestinal spasm; Gastrointestinal stinging; Gastrointestinal ulcer; Gastrointestinal ulcer management; Gastrointestinal ulcer NOS; Gastro-intestinal ulcer NOS; Gastrointestinal ulcer perforated NOS; Gastrointestinal ulcer perforation; Gastrointestinal ulcer perforation NOS; Gastrooesophageal junction ulcer; GDU; GI pain; Granulomatous stomatitis; Greater curvature gastric ulcer; Gum erosion; Gut pain; Haemorrhagic ulcer; Healed PU; Helicobacter duodenal ulcer; Hemorrhagic ulcer; Inflammation under tongue; Ischaemic ulcer; Ischemic ulcer; Lesser curvature gastric ulcer; Lip erosion; Mucosal prolapse syndrome; Mucosal ulceration; Mucosal ulceration NOS; Mucositis oral; Multiple gastric erosions; Multiple gastric ulcers; Oesophageal erosion; Oesophageal erosions; Oesophageal ulcer; Oesophageal ulcer perforation; Oesophageal ulceration; Oesophagitis ulcerative; Oesophagus ulceration; Pain peptic ulcer; Peptic ulcer; Peptic ulcer aggravated; Peptic ulcer complicated; Peptic ulcer disease; Peptic ulcer haemorrhagic; Peptic ulcer helicobacter; Peptic ulcer hemorrhagic; Peptic ulcer pain; Peptic ulcer perforated; Peptic ulcer perforation; Peptic ulcer perforation, obstructive; Peptic ulcer reactivated; Peptic ulcer repair; Peptic ulcer syndrome; Peptic ulcer type pain; Peptic ulcer uncomplicated; Peptic ulcer, obstructive; Peptic ulcer, site unspecified; Peptic ulceration; Peptic ulceration (healed); Perforated duodenal ulcer oversewing; Perforated duodenal ulcer repair; Perforated gastric ulcer; Perforated GU; Perforated peptic ulcer; Perforated peptic ulcer oversewing; Perforated peptic ulcer repair; Perforated stomach ulcer; Perforated ulcer; Perforated ulcer NOS; Perforation of prepyloric ulcer; Pharyngeal erosion; Prepyloric ulcer; Prophylaxis against drug-induced peptic ulcers; Prophylaxis against drug-induced ulcer; Prophylaxis against gastrointestinal ulcer; Prophylaxis NSAID gastropathy; Prophylaxis of NSAID gastric ulceration; PUD; Pyloric ulcer; Pyloric ulcer perforation; Pylorus ulcer; Reactivated duodenal

	ulcer; Reactivated GU; Reactivated peptic ulcer; Reactivated PU; Stomach erosion; Stomach ulcer; Stomach ulcer reactivated; Stomach ulcer with perforation; Stomal ulcer; Stomatitis; Stomatitis ulcerative; Stress ulcer; Stress ulcer aggravated; Stress ulcer bleeding; Syndrome peptic ulcer; Ulcer; Ulcer bleeding; Ulcer bleeding duodenal; Ulcer bleeding gastric; Ulcer bleeding peptic; Ulcer duodenal; Ulcer duodenal reactivated; Ulcer duodenal with perforation; Ulcer esophageal; Ulcer gastric; Ulcer gastroduodenal; Ulcer gastrointestinal; Ulcer NOS; Ulcer oesophageal; Ulcer of esophagus; Ulcer of oesophagus; Ulcer peptic; Ulcer peptic reactivated; Ulcer peptic with perforation; Ulcer prepyloric; Ulcer stomach; Ulcer stomach reactivated; Ulcer stomach with perforation; Ulcer stress; Ulcer syndrome peptic; Ulcer type pain; Ulceration; Ulcerative duodenitis; Ulcerative gastritis; Ulcerative stomatitis; Ulcerative stomatitis, acute; Ulcer-like dyspepsia; Ulcus ventriculi; Varioliform gastritis; Verrucous gastritis
Inflammation	Acute gastritis, with haemorrhage; Acute gastritis, with hemorrhage; Acute gastritis, without mention of haemorrhage; Acute gastritis, without mention of hemorrhage; Alcoholic gastritis, with haemorrhage; Alcoholic gastritis, with hemorrhage; Atrophic gastritis, with haemorrhage; Atrophic gastritis, with hemorrhage; Atrophic gastritis, without mention of haemorrhage; Atrophic gastritis, without mention of hemorrhage; Duodenitis, with haemorrhage; Duodenitis, with hemorrhage; Duodenitis, without mention of haemorrhage; Duodenitis, without mention of hemorrhage; Acute gastritis; Antral gastritis; Atrophic gastritis; Autoimmune gastritis; Bulbitis of duodenum; Catarrhal esophagitis; Catarrhal gastritis; Catarrhal oesophagitis; Chronic antral gastritis; Chronic duodenitis; Chronic erosive gastritis; Chronic gastritis; Chronic gastroduodenitis; Chronic nonatrophic gastritis; Corpus gastritis; Duodenitis; Enanthematic gastritis; Erosive duodenitis; Erosive esophagitis; Erosive gastroduodenitis; Erosive oesophagitis; Esophageal inflammation; Esophageal mucositis; Esophagitis; Esophagitis aggravated; Esophagitis ulcerative; Esophagitis, unspecified; Follicular gastritis; Fundal gastritis; Gastric inflammation; Gastric mucositis; Gastritis; Gastritis acute; Gastritis aggravated; Gastritis alcoholic haemorrhagic; Gastritis alcoholic hemorrhagic; Gastritis alkaline reflux; Gastritis atrophic; Gastritis atrophic haemorrhagic; Gastritis atrophic hemorrhagic; Gastritis bile acid; Gastritis biliary; Gastritis chronic; Gastritis erosive; Gastritis haemorrhagic; Gastritis haemorrhagic aggravated; Gastritis hemorrhagic; Gastritis hemorrhagic aggravated; Gastritis hypertrophic; Gastritis NOS; Gastritis prophylaxis; Gastroduodenitis; Gastroesophagitis; Gastrointestinal inflammation; Gastrointestinal mucositis; Gastrooesophagitis; Granulomatous gastritis; Granulomatous stomatitis; Haemorrhagic erosive gastritis; Haemorrhagic gastritis; Hemorrhagic erosive gastritis; Hemorrhagic gastritis; Inflammation stomach; Inflammation under tongue; Oesophageal inflammation; Oesophageal mucositis; Oesophagitis; Oesophagitis aggravated; Oesophagitis NOS; Oesophagitis ulcerative; Oesophagitis, unspecified; Other esophagitis; Other oesophagitis; Pangastritis; Peptic duodenitis; Phlegmonous gastritis; Reflux gastritis; Stomach inflammation; Stomatitis; Superficial gastritis; Superficial gastroduodenitis; Type A gastritis; Type B gastritis; Type C gastritis; Ulcerative gastritis; Varioliform gastritis; Verrucous gastritis; Worsening of esophagitis; Worsening of oesophagitis
